# A generalized reinforcement learning based deep neural network agent model for diverse cognitive constructs

**DOI:** 10.1038/s41598-023-32234-y

**Published:** 2023-04-12

**Authors:** Sandeep Sathyanandan Nair, Vignayanandam Ravindernath Muddapu, C. Vigneswaran, Pragathi P. Balasubramani, Dhakshin S. Ramanathan, Jyoti Mishra, V. Srinivasa Chakravarthy

**Affiliations:** 1 Computational Neuroscience Lab, Department of Biotechnology, Bhupat and Jyoti Mehta School of Biosciences, Indian Institute of Technology Madras, Room 505, Block 1, Sardar Patel Road, Adyar, Chennai, Tamil Nadu 600036 India; 2 Neural Engineering and Translation Labs, Department of Psychiatry, University of California, San Diego, La Jolla, CA USA; 3 Department of Mental Health, VA San Diego Medical Center, San Diego, CA USA; 4 Present Address: Blue Brain Project, École Polytechnique Fédérale de Lausanne (EPFL), Campus Biotech, 1202 Geneva, Switzerland; 5 Present Address: Department of Cognitive Science, Indian Institute of Technology, Kanpur, Kanpur, India

**Keywords:** Neuroscience, Computational neuroscience

## Abstract

Human cognition is characterized by a wide range of capabilities including goal-oriented selective attention, distractor suppression, decision making, response inhibition, and working memory. Much research has focused on studying these individual components of cognition in isolation, whereas in several translational applications for cognitive impairment, multiple cognitive functions are altered in a given individual. Hence it is important to study multiple cognitive abilities in the same subject or, in computational terms, model them using a single model. To this end, we propose a unified, reinforcement learning-based agent model comprising of systems for representation, memory, value computation and exploration. We successfully modeled the aforementioned cognitive tasks and show how individual performance can be mapped to model meta-parameters. This model has the potential to serve as a proxy for cognitively impaired conditions, and can be used as a clinical testbench on which therapeutic interventions can be simulated first before delivering to human subjects.

## Introduction

High-level human cognition consists of a variety of functions or capabilities, including selective processing of goal-relevant information, suppression of goal-irrelevant information, action selection, reward processing, working memory, etc. There is a long history of empirical research that studies the various cognitive functions individually while excluding other functions. However, to understand these cognitive functions as functions of an integrative agent, it is essential to study them holistically, revealing the synergies among these functions that come into play as an agent interacts with its environment.

While empirical research on cognitive functions suffers from this fragmented approach due to several challenges including participant burden, limited resources and expertise, theoretical investigation also often reflects this piecemeal approach, offering a wide variety of models that describe individual cognitive functions. There have been efforts to construct integrative computational frameworks that capture a range of cognitive functions. For example, the “ACT-R” system has been proposed as a general framework for modeling a wide variety of cognitive processes^1^. Subsequently, it was extended to include visual attention, and its properties, like speed and selectivity, as they vary from subject to subject^1^. Similarly, the “Soar” architecture can successfully integrate different levels of reasoning, planning, reactive execution, and learning from experience^2^. The importance of more holistic and integrative models has been emphasized by several researchers, resulting in many unified computational models of cognition. As these models evolved, a certain similarity among these modeling architectures began to reveal itself. For example, common features of three such cognitive architectures viz., ACT-R^1^, Soar^2,3^, and SIGMA^4^ have been described^5^. More on fragmented and integrative approach is updated in the supplementary material.

Neuropsychiatric disorders are characterized by a wide range of cognitive dysfunctions, and the degree to which these disorders are mapped to specific neural substrates is still being resolved^6^. Just as theoreticians sought to create unified architectures of cognition, experimental cognitive scientists also made efforts to move away from exclusivist approaches and began to study multiple cognitive functions in human population cohorts simultaneously^7^. One such experimental system is the *BrainE* platform, which includes a range of cognitive assessments like selective attention (SA), response inhibition (RI), working memory (WM), and distractor processing (DP) in both non-emotional and emotional context^8^. The *BrainE* system measures both behavioral parameters and electroencephalography (EEG) signals, thereby creating an opportunity to relate cognitive behavior to neural substrates.

In this paper, we present a unified architecture of cognition that can model a range of cognitive functions, including SA, RI, WM, DP, etc. The proposed model has the following components: 1) sensory representation, 2) memory, 3) value computation, 4) exploration, and 5) action selection. The model is cast broadly within the framework of reinforcement learning (RL)^9–11^. Notably, the action selection strategy, which involves pursuit of explorative and exploitative modes, each of which is regulated based on the underlying value dynamics is novel to our approach. The model has elements common to deep neural networks and two novel neural elements that are not typically found in such networks viz., 1) flip-flop neurons and 2) oscillator neurons. First proposed in^12^, the flip-flop neurons are fashioned after flip-flops in digital systems theory and can store memories. In the oscillator network, the lateral interactions are designed such that the oscillators exhibit desynchronized dynamics. Such oscillator networks have been used before to implement exploratory functions essential for achieving randomness in action selection in RL models^13^. We hypothesized that this modeling framework can replicate the subject's performance with respect to diverse cognitive decision-making tasks.

In the following section, we will describe the methods starting with a brief overview of the experimental setup, the cognitive testing paradigms used, and the various tasks conducted to evaluate cognitive abilities. This is followed by a brief overview of the model architecture that is used to simulate the experimental tests, various building blocks of the model architecture and their mathematical formulation, and how they are integrated to mimic an experimental subject. In the “Results” section, we summarize the main results, including the training phase, the performance evaluation and the meta-parameters used for tuning the performance. We compare the model performance with experimental results. The final section concludes the results and discusses the utility, limitations, and future scope.

## Materials and methods

**Figure 1:**
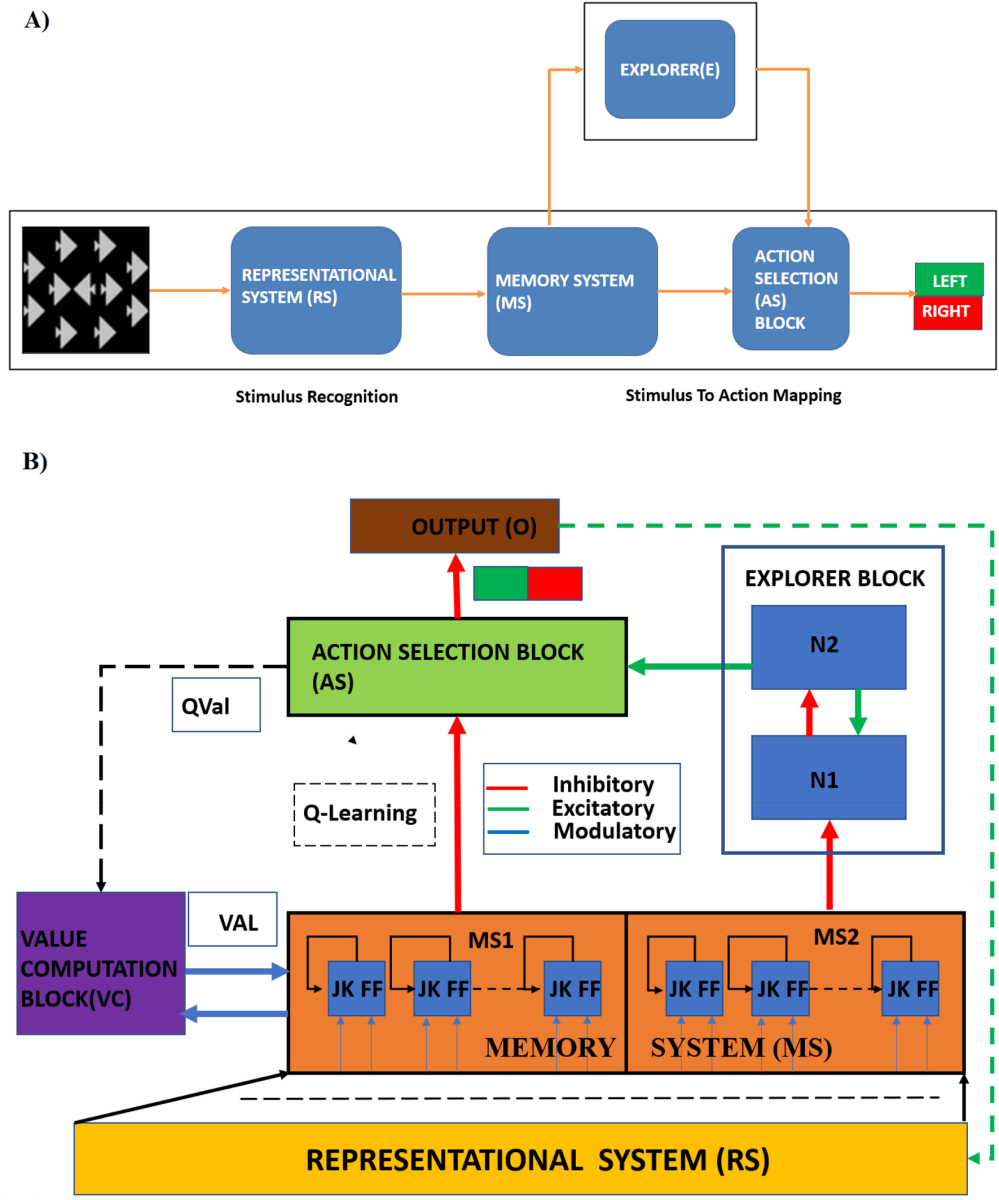
(**A**) Overview of (Generalized Reinforcement Learning-based Deep Neural Network) GRLDNN model architecture. RS, Representational System is used for stimulus recognition; Memory System (MS) and Action Selection (AS) block along with the Explorer (E) is used for stimulus to action mapping. The encoder output from RS is presented to the MS where the stimulus is processed, and the action selection takes place at AS. (**B**) Block diagram of the Agent Model architecture. RS, Representation System Block; Explorer Block consisting of N1/N2 Oscillator pair; AS, Action Selection Block; O, Output block; VAL, Value Computation Block where the Value function is computed; QVal, Q-value function; MS, Memory System block.

The proposed Generalized Reinforcement Learning-based Deep Neural Network (GRLDNN) agent model, as shown in the Fig. 1, can simulate various experimental paradigms that can test different cognitive functions such as SA, RI, WM, and DP.

### The experimental tasks

A repertoire of tasks was prescribed to capture a range of cognitive functions that comprise human decision-making. The cognitive functions that we focus on in these tasks are the ability to selectively attend to relevant stimuli, inhibit responses to irrelevant stimuli, avoid distractors during an attention task, and working memory. We primarily focused on modeling the cognitive assessments like SA, RI, WM, DP conducted using *BrainE* platform^8^. In addition to this we have also modeled additional experimental paradigms such as the N-back task to assess working memory load and the 2 × 5 task, which evaluates the sequence processing capabilities. To test the effectiveness of the Q-learning network we have also modeled the T-maze and Grid-world tasks. The details of the tasks are described in the supplementary material.

### GRLDNN (Generalized reinforcement learning-based deep neural network) agent model

We present a unified RL-based deep network architecture that can simulate all the experimental tasks described in Supplementary material (Section S1).

A schematic of the model architecture is given in Fig. 1. The model has five distinctly identifiable components viz.—1) Representational System (RS) consists of a series of layers—convolutional layers followed by fully-connected layers, that generate compact representations of the input images. 2) Memory System (MS) is a layer of flip-flop neurons that receives the inputs from the RS via a fully connected weight stage. This system has the memory property. 3) Value Computation System (VC) combines the neural outputs of the MS and computes the value function. 4) Explorer comprises a nonlinear oscillator network, wherein the oscillators interact via inhibitory connections, generating desynchronized oscillations. The randomness inherent in the chaotic oscillatory dynamics of this system introduces a level of randomness in the action selection at the output layer, driving exploratory behavior. 5) Action Selection System (AS) is the output of the entire architecture that combines the outputs of the MS via a trainable weight stage and the output of the Explorer. The AS is trained using Q-learning described in detail in the supplementary materials^14^.

#### Representational system

The input stimulus is presented as an image to the input layer of the RS, which is trained as a convolutional autoencoder^15,16^. The RS module consists of an input layer followed by four convolutional and max-pooling layers. A fully connected layer follows the four layers of the convolutional and max-pooling layers. Another fully connected later is used to reduce the encoder output to 1 × 64. The convolutional layers use a 3 × 3 filter window size. Mean squared error is used as the output loss. At the decoder end, the 1 × 64 feature output is expanded, followed by deconvolutional layers and pooling layers, at the end of which the original image is reconstructed back. Output from the fully connected layer of the encoder part of RS is provided as the input to the MS.

#### Memory system

The output of the RS module is presented to the MS via a fully connected weight stage. The MS, as mentioned before, is a 1D layer of flip-flop neurons. This layer is divided into two equal sections—MS1 and MS2. MS1 has D1-type flip-flop neurons, whereas MS2 has D2-type flip-flop neurons. The flip-flop architecture is provided in the supplementary material.

The feature vector from the RS module ($$X_{RS} )$$ reaches the flip-flop neurons of MS1 and MS2 over the weight stages ($$W_{i}^{RS \to MS1} )$$ and $$W_{i}^{RS \to MS2}$$, respectively. So, the effective input received at MS1 is $$W_{i}^{RS \to MS1} \left( t \right)*X_{RS}$$ (Supplementary material).

The output of the encoder part of the RS module is presented as the input, $X_{RS} \left( t \right),$ to the J and K ports of the flip-flop neurons present in the MS1/MS2 sub-blocks of the MS, $W_{i}^{RS \to JMS1} , W_{i}^{RS \to KMS1} ,$ $W_{i}^{RS \to JMS2}$ and $W_{i}^{RS \to KMS2}$ denote the weights from the RS layer to the respective J and K inputs of the MS1/MS2 sub-blocks (Supplementary material).

##### Computations in the MS1/MS2 sub-blocks of the memory system

The J and K inputs for the flip-flop neurons of MS1 and MS2 are given by Eqs. (1,2,3,4) below. The output of the flip-flop neuron is expressed by Eqs. (5, 6), which is in line with the circuit diagram and the truth table given in the supplementary material. The output is also influenced by the modulatory input received from the VC^17^.

1 $$J_{MS1} (t) = W_{i}^{RS \to JMS1} \left( t \right)X_{RS} \left( t \right)$$

2 $$K_{MS1} \left( t \right) = W_{i}^{RS \to KMS1} X_{RS} \left( t \right)$$

3 $$J_{MS2\left( t \right)} = W_{i}^{RS \to JMS2} \left( t \right)X_{RS} \left( t \right)$$

4 $$K_{MS2} \left( t \right) = W_{i}^{RS \to KMS2} X_{RS} \left( t \right)$$

5 $$V_{i}^{MS1} \left( t \right) = J_{MS1} \left( {1 - V_{i}^{MS1} \left( {t - 1} \right)\lambda_{MS1} } \right) + \left( {1 - K_{MS1} } \right)V_{i}^{MS1} \left( {t - 1} \right)\lambda_{MS1}$$

6 $$V_{i}^{MS2} \left( t \right) = J_{MS2} \left( {1 - V_{i}^{MS2} \left( {t - 1} \right)\lambda_{MS2} } \right) + \left( {1 - K_{D2} } \right)V_{i}^{D2} \left( {t - 1} \right)\lambda_{D2}$$

7 $$V_{i}^{MS1} \left( t \right) = \lambda^{MS1} \left( t \right)W_{i}^{RS \to MS1} \left( t \right)X_{RS} \left( t \right)$$

8 $$V_{i}^{MS2} \left( t \right) = \lambda^{MS2} \left( t \right)W_{i}^{RS \to MS2} \left( t \right)X_{RS} \left( t \right)$$

9 $$\lambda^{MS1} \left( t \right) = \left( {\frac{1}{{1 + e^{{K_{1} *\left( {VAL\left( t \right) - \theta_{MS1} } \right)}} }}} \right)$$

10 $$\lambda^{MS2} \left( t \right) = \left( {\frac{1}{{1 + e^{{K_{2} *\left( {VAL\left( t \right) - \theta_{MS2} } \right)}} }}} \right)$$

where $K_{1} < 0$ and $K_{2} > 0$ and $\lambda^{MS1} and \lambda^{MS2}$ are the sigmoid gain parameters.

#### Value computation system

The value is computed using a weighted sum of the outputs of the flip-flop neurons of MS1. Thus, the value function ‘VAL’, is computed as per Eq. (11) below,

11 $$VAL\left( t \right) = \mathop \sum \limits_{i = 1}^{n} W_{i}^{MS1 \to VC} \left( t \right)V_{i}^{MS1}$$

#### Explorer module

The explorer module consists of a network of nonlinear oscillators. These are thought to be implemented by two pools of neurons, N1 and N2, connected back-to-back. The MS2-type flip-flop neurons of MS project to N1, whereas the output of the N1 neural layer, in turn, influences the N2 neural layer. N1 and N2 form a loop, with inhibitory projections from N1 to N2 and excitatory projections from N2 to N1. Such excitatory-inhibitory pairs of neurons pools have been shown to exhibit oscillations^10,18^. In the present case, it turns out that the equations that couple a single N1 neuron bidirectionally to a single N2 neuron can be classified as a general oscillator system called Lienard system, which exhibits limit cycle oscillations^19^. The dynamics of N1-N2 neuronal pools is defined as,

12 $$\tau_{N1} \frac{{dV_{i}^{N1} }}{dt} = - V_{i}^{N1} + \mathop \sum \limits_{j = 1}^{n} W_{ij}^{N1 \to N1} V_{j}^{N1} + W_{i}^{N2 \to N1} V_{i}^{N2} - V_{i}^{MS2} \left( t \right)$$

13 $$\tau_{N2} \frac{{dU_{i}^{N2} }}{dt} = - U_{i}^{N2} + \mathop \sum \limits_{j = 1}^{n} W_{ij}^{N2 \to N2} V_{j}^{N2} \left( t \right) - W_{i}^{N1 \to N2} V_{i}^{N1}$$

14 $$V_{i}^{N2} \left( t \right) = \tanh \left( {\lambda^{MS2} \left( t \right)U_{i}^{N2} } \right)$$

15 $$V_{j}^{A} \left( t \right) = \mathop \sum \limits_{i = 1}^{n} W_{i,j}^{MS1 \to AS} \left( t \right)V_{i}^{MS1}$$

16 $$V_{j}^{B} \left( t \right) = \mathop \sum \limits_{i = 1}^{n} W_{i,j}^{N2 \to AS} \left( t \right)V_{i}^{N2} \left( t \right)$$

where $V_{i}^{N1}$ and $U_{i}^{N2}$ are the internal states of N1 and N2 neurons, respectively, $V_{i}^{N2}$ is the output of the N2 neuron, $W^{N1 \to N1}$ and $W^{N2 \to N2}$ are weight kernels representing lateral connectivity in N1 and N2 modules, respectively, $\tau_{N1}$ and $\tau_{N2}$ are the time constants of N1 and N2, respectively, $W^{N1 \to N2}$ is the connection strength from N1 to N2, $W^{N2 \to N1}$ is the connection strength from N2 to N1, and $\lambda_{N2}$ is the parameter which controls the slope of the sigmoid in N2. $V_{j}^{A} \left( t \right) \;and\; V_{j}^{B} \left( t \right)$ are the inputs arriving at AS1 block from MS1 and MS2 (via N1 &N2) respectively.

##### Lateral connections among coupled oscillators of the explorer module

The lateral connectivity in the N1 or N2 network is modeled using constant weights where weights between two neurons is given by $W^{N1 \to N1}$ and $W^{N2 \to N2}$,

17 $$\begin{aligned} & - W_{ij}^{N1 \to N1} = W_{ij}^{N2 \to N2} = 1 \;for\; i = j; \\ & - W_{ij}^{N1 \to N1} = W_{ij}^{N2 \to N2} = \epsilon , \;otherwise ; \\ \end{aligned}$$

where $\epsilon$ is the magnitude of the connection strength of lateral connections among the neighbouring neurons in both N1 and N2 modules.

#### Action selection module: Q-learning

The network's final output is a linear sum of the output of the MS1 block and the output of the explorer module.

18 $$V_{i}^{A} \left( t \right) = \mathop \sum \limits_{i = 1}^{n} W_{i,j}^{MS1 \to AS} \left( t \right)V_{i}^{MS1}$$

The output $V_{i}^{A}$ is combined with the output $V_{i}^{B}$ (Eq. 16) at the AS block as shown in (Eq. 19) below.

19 $$\tau_{AS} \frac{{dV_{i}^{AS} }}{dt} = - V_{i}^{AS} - V_{i}^{A} \left( t \right) + V_{i}^{B} \left( t \right)$$

20 $$\tau_{AS} \frac{{dV_{i}^{AS} }}{dt} = - V_{i}^{AS} - \lambda^{MS1} \left( t \right) V_{i}^{MS1} \left( t \right) + \lambda^{MS2} \left( t \right) W_{i}^{N2 \to AS} V_{i}^{N2} \left( t \right)$$

The action selection mechanism at AS is facilitated using a race model^20–23^. The output of the AS block (− $$V_{i}^{AS} )$$ is compared against a threshold $$Vth$$. The neuron (ith) whose output crosses the threshold first, is considered a winner, and the $$ith$$ action is selected.

21 $$If, V_{i}^{AS} \left( t \right) > V_{th} ;\;then\; AS = i$$

##### Reward and learning

The weights between the neurons in the MS1 block and the AS module are updated using Q-Learning^24^ as shown in (Eqs. 22,23,24).

22 $$Q_{t} \left( {s,a} \right) = V_{j}^{N1} \left( t \right)$$

23 $$\delta \left( t \right) = r\left( t \right) + \gamma \mathop {\max }\limits_{a} Q_{t + 1} \left( {s,a} \right) - Q_{t} \left( {s,a} \right)$$

where $\gamma = 0$ (discount factor).

24 $$Q_{t + 1} \left( {s,a} \right) = Q_{t} \left( {s,a} \right) + \eta \left( {r\left( t \right) + \gamma \mathop {\max }\limits_{a} Q_{t + 1} \left( {s,a} \right) - Q_{t} \left( {s,a} \right)} \right)$$

#### Backward propagation

In this model, trainable weights are located in three areas: i) MS1-block to AS weight stage, ii) MS1 block to VC block weight stage, and iii) RS to MS1/MS2 block weight stage (Supplementary material). The weights between the various modules are updated using the backpropagation algorithm. The weight update between the MS1 and the AS blocks is governed by Q-learning, while the weight update between MS1 to VC is done using temporal difference (TD)-learning. The MS1/MS2 subblocks used flip-flop neurons, and the weight update between RS and MS is done using TD-learning. The weight training equations are described in detail in the supplementary material.

More details about the Markov decision model and the state and action spaces are given in the supplementary material.

### Performance assessment

The model performance is assessed in terms of the metrics of accuracy, reaction time, speed, consistency, and efficiency, as defined in Supplementary material. We have also.

## Results

In this section, we describe the performance of the GRLDNN model that is used to simulate the four cognitive functions of SA, RI, WM, and DP experimentally assessed using the *BrainE* platform. In the subsequent subsections, we will first show the progress of state value functions and the Q-value functions during the learning process. Then we will show the impact of parameter tuning with respect to lateral connections strengths and threshold ($\epsilon$ and $V_{TH}$). Then we implement an inverse model using a neural network that can calibrate the meta parameters of the GRLDNN model so that the model can simulate the experimental performance. We then present a comparison of the average performance between both the model and the experimental results. In addition to the results of the cognitive assessments conducted using *BrainE* platform^8^, we also show the performance results of the N-back task, which tests the working memory load and present a comparison between the model and the experimental results. The results of the other tasks such as the 2 × 5(sequence processing) and T-maze are updated in the supplementary material.

### Training phase

**Figure 2:**
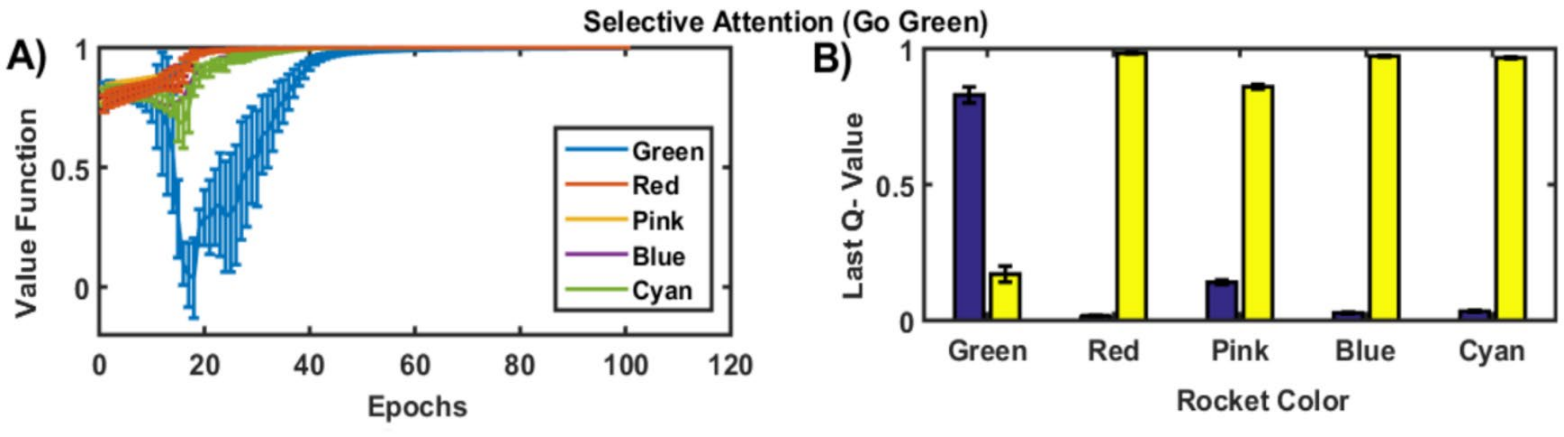
(**A**) The state value functions over the epochs during the training phase of the Go Green (SA) task. (**B**) The Q-value at the end of training for the Go Green task that requires selective attention to the green colored rockets while ignoring other isoluminant colors—red, pink, blue, cyan. The blue bar represents the ‘Go’ action and the yellow bar represents the ‘No-Go’ action.

As the learning progresses, the magnitude of the value functions at the end of each trial keeps increasing and approach the maximum value of 1, as the model learns the task accurately. The Q-values for the respective state and action pairs corresponding to the correct action are higher, whereas the values corresponding to the wrong action are lower. Figure 2A represents the state value function for the *Go Green* (SA) task, and Fig. 2B represents the Q-value at the end of training epochs, at the last instance of the *Go Green* trials. The state value and Q-value functions for other tasks are described in the supplementary material.

The test dataset contains 33% of the green-colored rockets and 67% of other colored rockets in the SA task. The blue bar represents the ‘Go’ action and the yellow bar represents the ‘No Go’ action.

### Effect of parameter tuning

The performance of cognitive tasks depends on various factors. We observed variations in the model performance by tuning specific meta parameters, using performance metrics of accuracy, reaction time, speed, consistency, and efficiency. The meta-parameters that are varied are $\epsilon$ and $V_{TH}$.

- $\epsilon$ influences the *lateral connectivity strength* of the N1 and N2 coupled oscillator system, which controls the level of exploration in the model.
- $V_{TH}$ is the *threshold* that appears in the race model, used in the AS module where the action selection occurs, which controls how fast the decision can be made (reaction time).

#### Effect of the threshold (***V***_***TH***_) and lateral connectivity strength ($\epsilon$) on the performance

**Figure 3:**
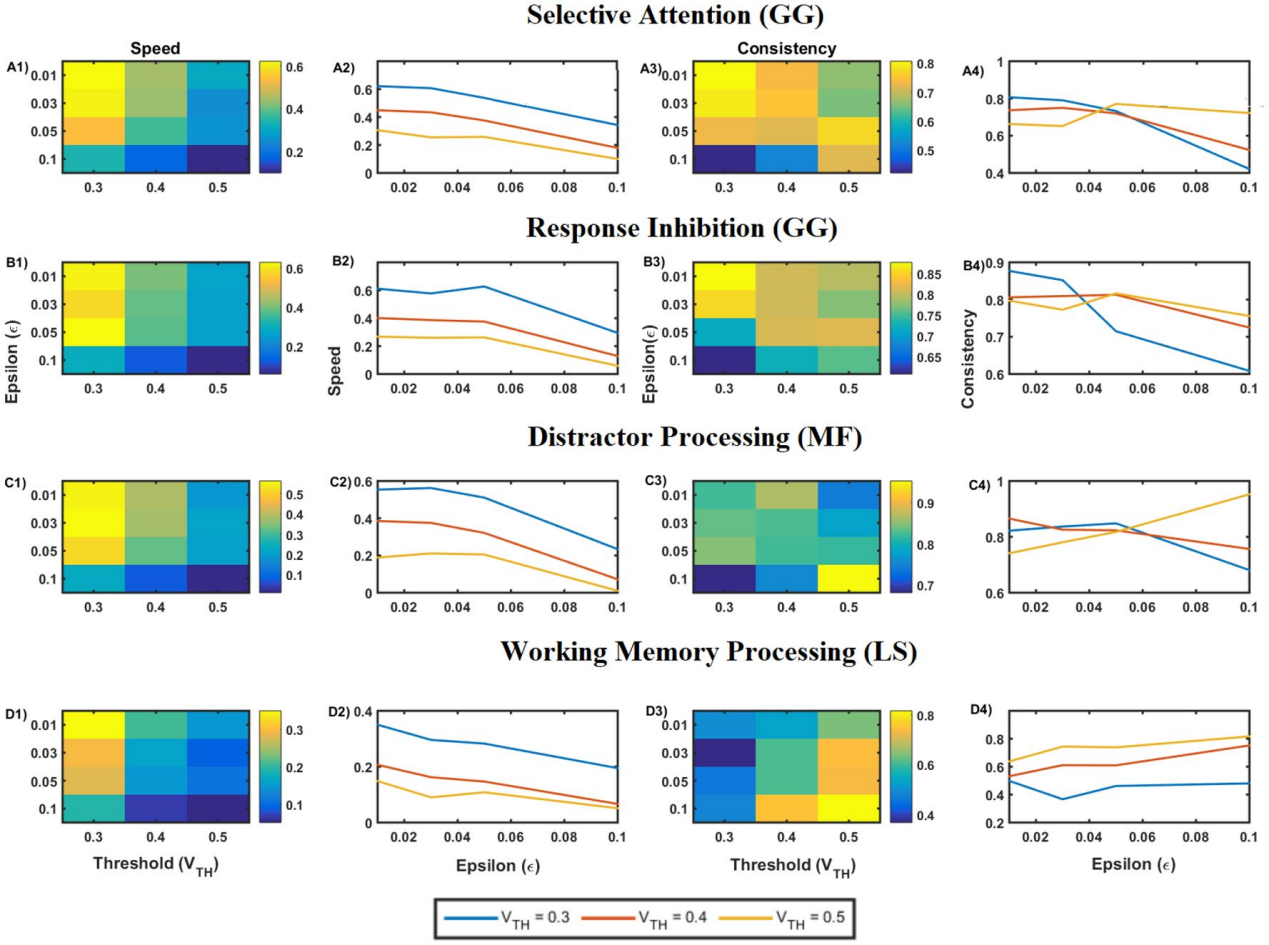
Plot of performance parameters for various tasks, with threshold ( varying between 0.3 to 0.5 and lateral connection strength $\epsilon$ ranging between 0.01 to 0.1. (**A1**,**A2**) The speed with which the decision is made for Go-Green (SA) task, (**A3**,**A4**) Consistency for Go-Green (SA) task, which indicates how consistent the performance was with respect to speed across trials. It also indirectly indicates the standard deviation of the speed of performance. Similarly, (**B1**–**B4**) represents the Go-Green (Response Inhibition) task, (**C1**–**C4**) indicates the Middle Fish (Distractor Processing) task, (**D1**–**D4**) indicate performance on the Lost Star task.

Figure 3 shows the variation in performance with respect to speed and consistency for various values of lateral connection strength ($\epsilon$) and threshold (*V*_*TH*_) illustrated for Go Green (SA) task. *V*_*TH*_ is varied in the range of 0.3 to 0.5 in steps of 0.1 and $\epsilon$ takes values of 0.01, 0.03, 0.05 and 0.1. Figure 3A1 and Fig. 3A2 show the variation in decision-making *speed* with respect to changes in $\epsilon$ and *V*_*TH*_. Figure 3A3 and Fig. 3A4 shows a similar variation in *consistency*. From the results, it can be seen that speed is inversely proportional to both the threshold and lateral connection strength.

Our results show that for lower values of the action selection threshold, the model performance exhibit higher speeds and vice versa. This is expected since the lower the threshold, the faster the action selected. The AS block being implemented as a race model decides on the action to be selected based on the threshold.

**Figure 4:**
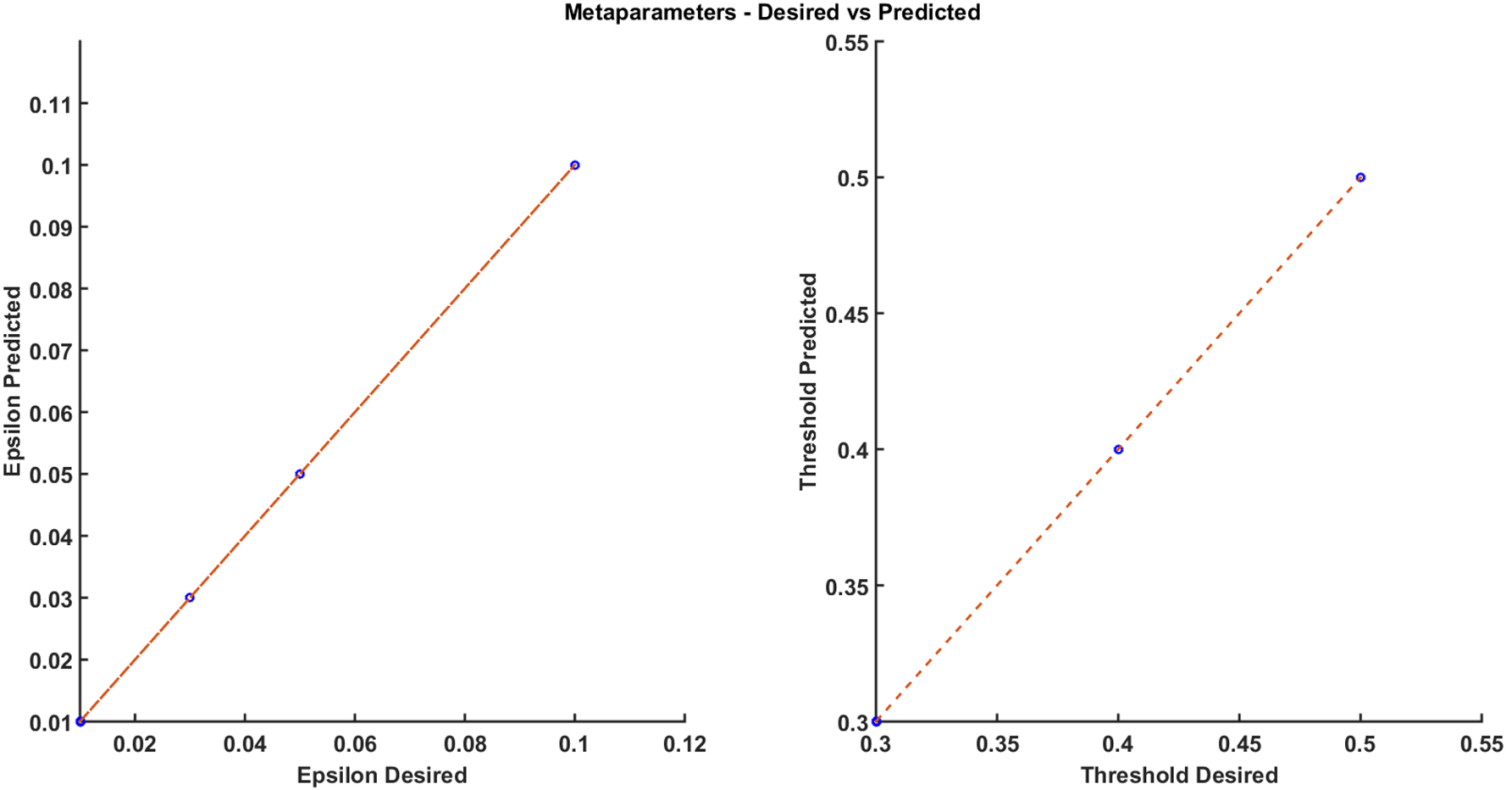
(**A**) The parameter fit is checked for the predicted vs. desired meta parameter ($\epsilon$). (**B**) The parameter fit is checked for the predicted vs. desired meta parameter (V_TH_).

Hence by searching for an optimum in the meta-parameter space consisting of lateral connection strength and action selection threshold ($\epsilon ,\;V_{TH}$), it is possible to match the model's performance with experimental data. We modeled the mapping between the experimental parameters (speed, consistency) and the model meta-parameters ($\epsilon ,\;V_{TH}$), tuned using a simple multilayer perceptron model (MLP). Given the input values of speed and consistency, we can predict the corresponding values of $\epsilon$ and $V_{TH}$. Hence by varying the values of the two meta-parameters, we were able to calibrate the GRLDNN model so that the model can approximately simulate the experimental performance. The model fit between the predicted and desired values of $\epsilon$ and $V_{TH}$ is as shown in Fig. 4 below for the *Go Green* (SA) task. Twelve data points were used for training from the combination of meta-parameters ($\epsilon$ and $V_{TH}$), and the training error was in the order of ~ 10^−5^ after 50,000 epochs. The predicted and desired values of both the threshold and epsilon were matched.

The performance results of the GRLDNN agent model were compared with the experimental results of healthy subjects^8^. In the GRLDNN agent model, decision-making scenarios were simulated for the different cognitive functions of SA, RI, WM, DP by modeling the test paradigms for *Go Green*, *Middle Fish*, and *Lost Star* tasks. The model's performance was tuned using the meta-parameters ($V_{{TH\left( {pred} \right)}} \;and\;\epsilon )$ to match the experimental results. By navigating through the parameter space as shown in Fig. 3, we selected the values of $V_{TH} = 0.4 \;and\;\epsilon = 0.05$, which was found to be closely matching with the average performance results of the experimental subjects. The RMSE (root mean squared error) was found to be lowest at 0.007 for $V_{TH} = 0.4 \;and\;\epsilon = 0.05$ when compared with the average performance of experimental results.

**Figure 5:**
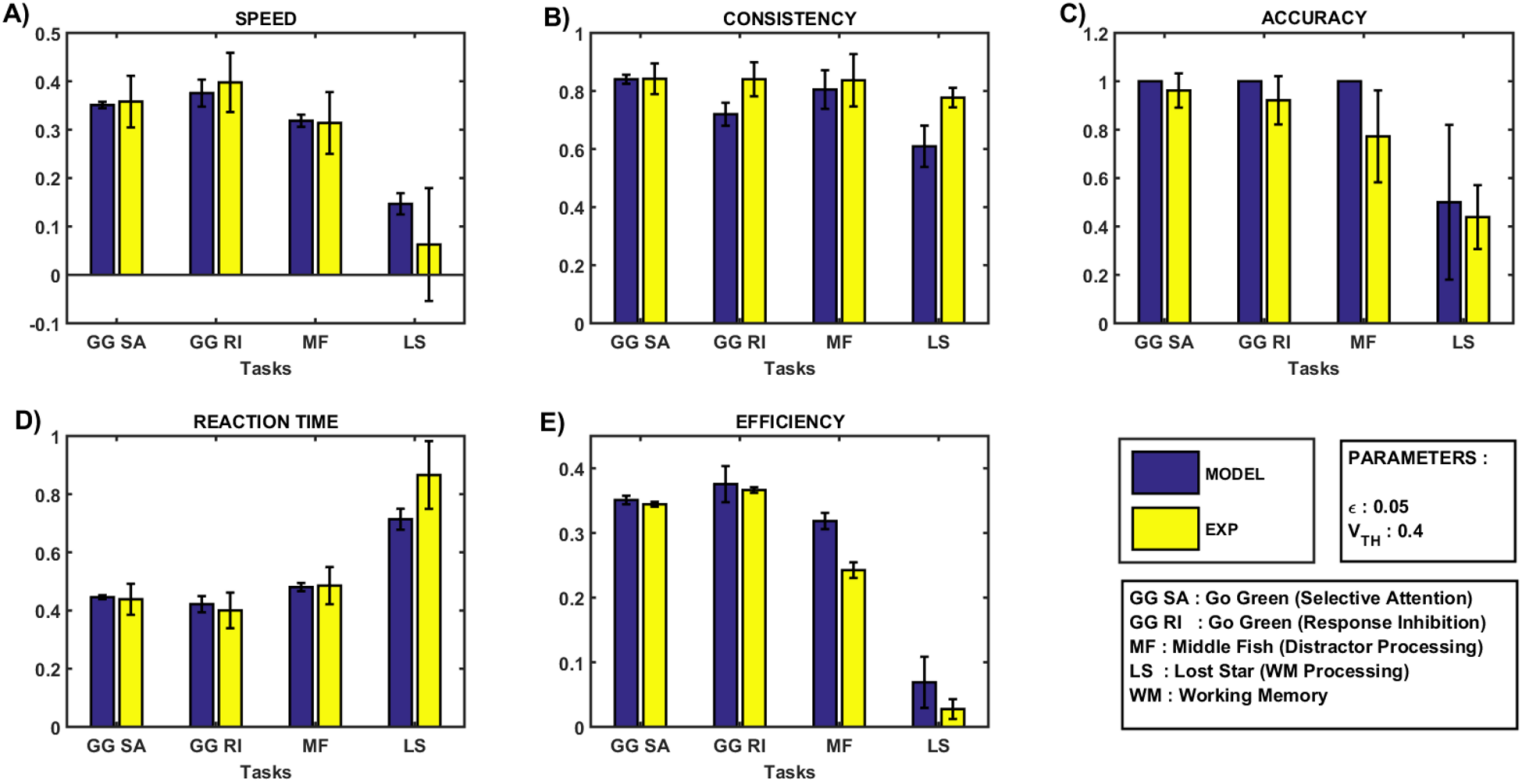
Comparison of performance of the GRLDNN model with the experimental results and data adapted from^8^ (**A**) Speed (**B**) Consistency, (**C**) Accuracy, (**D**) Reaction Time, (**E**) Efficiency. EXP, experimental results; MODEL, Model performance Results.

Figure 5 shows the performance comparison of the model and experimental data, where the blue bar represents the model performance, and the yellow bar represents the experimental performance results for all modeled tasks. As seen in the case of the *Go Green* task, the GRLDNN agent model recorded an average speed of $0.3510 \pm 0.065 { }$ and $0.3756 \pm 0.0279$ for SA and RI, respectively (Fig. 5A, dark blue bar) compared to the experimental results, which recorded an average speed of $0.3580 \pm 0.0534$ and $0.3976 \pm 0.0612$, for SA and RI, respectively (Fig. 5A, yellow bar). Similar comparisons can be made for performance metrics for all modeled tasks.

Hence by mapping the performance characteristics across different cognitive tasks onto the meta-parameter space and navigating through the same, we are able to replicate empirical performance on different cognitive abilities using the GRLDNN model.

**Figure 6:**
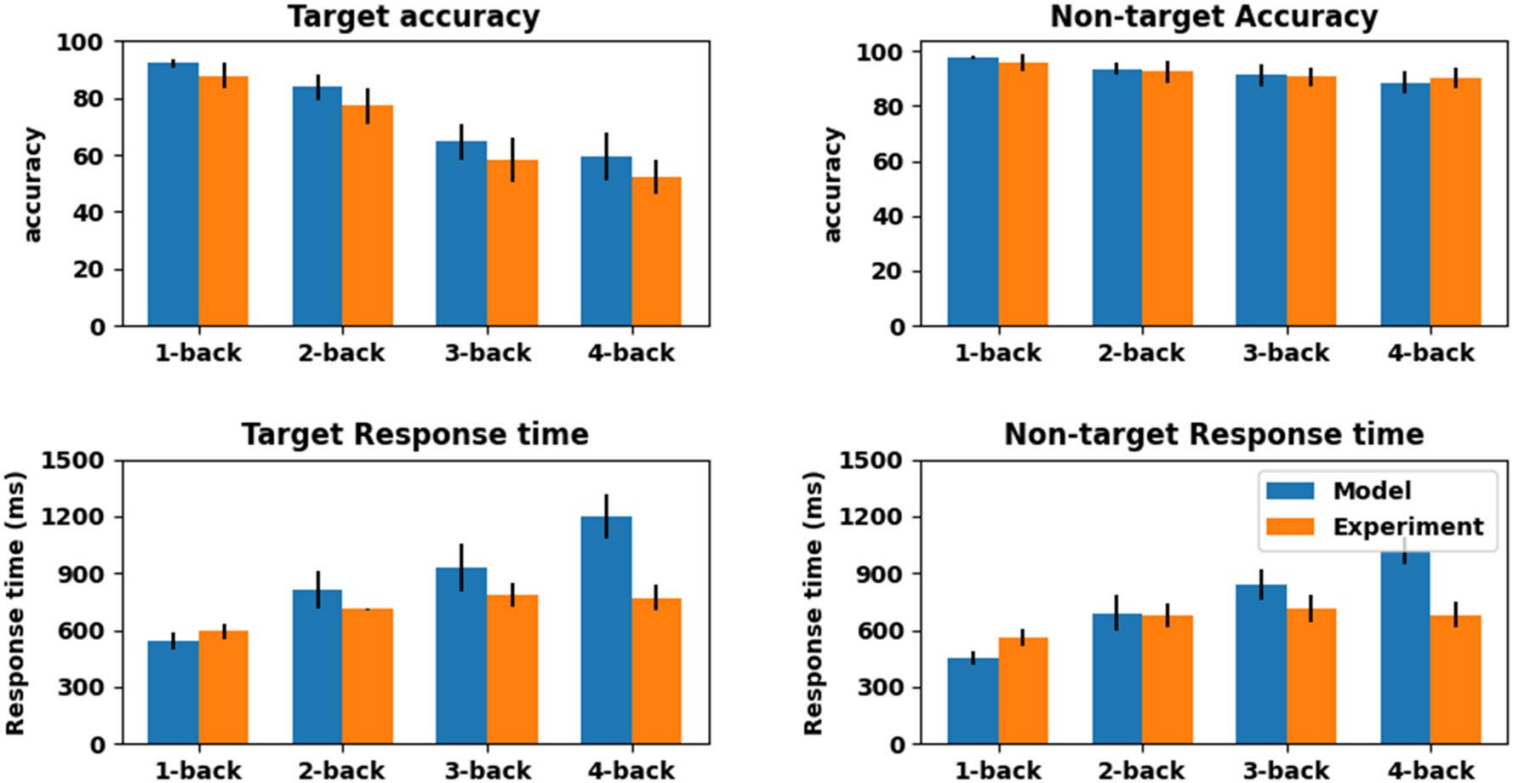
Comparison of performance of the GRLDNN model with the experimental results and data adapted from^25^ (**A**) Accuracy when the current stimulus is the target (**B**) Accuracy for non-target stimulus , (**C**) Response time for Target Stimulus, (**D**) Response Time for non-Target Stimulus.

The performance characteristics of the N-back task is as shown in Fig. 6. The model performance results of the N-back task are comparable and relatable to the experimental results^25^. The response time and the accuracy were evaluated for both target and non-target stimuli. We have simulated up to N = 4. The blue bar indicates the model performance and the orange bar indicates the experimental results.

The performance characteristics of the sequence processing (2 × 5) and other tasks are updated in the supplementary material. We have considered two additional RL tasks that involves action based state transitions to show the effectiveness of our model. We have also discussed the stability aspects and convergence of the Q networks in the supplementary material.

## Discussion

Using the proposed GRLDNN model, we successfully modeled tasks to assess a variety of cognitive abilities, including SA, RI, WM, and DP. By varying the meta-parameters, $V_{TH} \;and \;\epsilon ,$ we were able to tune the performance outputs of the model (Fig. 3). Notably, our model results were comparable to that of experimental results for healthy subjects, as shown in Fig. 5.

**Figure 7:**
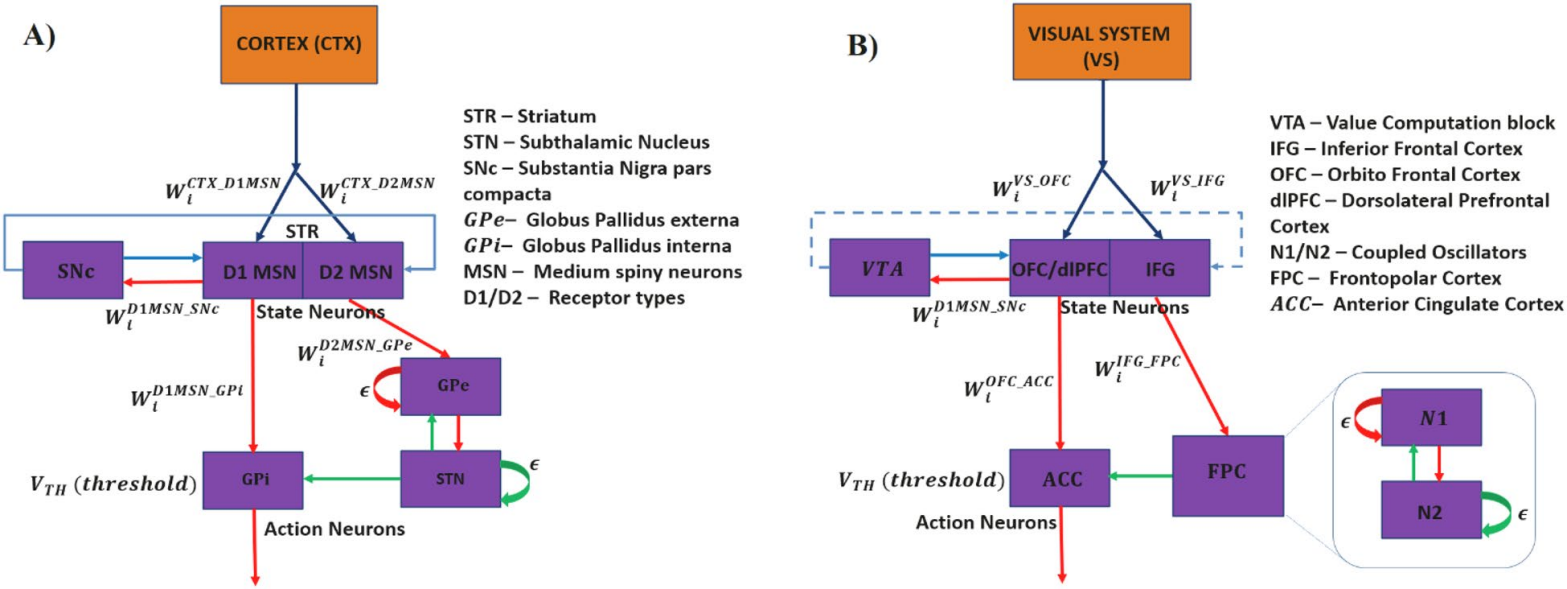
Biological Representation of GRLDNN Agent Model inspired by (**A**) Basal Ganglia (BG) architecture and (**B**) the equivalent representation of prefrontal cortex (PFC) Architecture. SNc, Substantia Nigra pars compacta; STR, Striatum; GPi, Globus Pallidus interna; GPe, Globus Pallidus externa; STN, Subthalamic Nucleus; MSN, Medium Spiny neurons; STN-GPe forms a coupled oscillator system with interconnectivity weights W between STN and GPe and $\epsilon$ is the lateral connection strengths among the neurons of STN and GPe. Action is selected based on the winner neuron crossing the threshold (V_TH_) first. VTA, ventral tegmental area; OFC, Orbitofrontal cortex; DLPFC, dorsolateral prefrontal cortex; IFG, inferior frontal gyrus; FPC, frontopolar cortex; ACC, anterior cingulate cortex; FPC is modeled using a coupled oscillator system with interconnectivity weights W between oscillators N1 and N2 and $\epsilon$ is the lateral connection strengths among the neurons of N2. OFC/DLPFC to ACC weights are updated using Q-learning.

The current GRLDNN model is an agent model built using a reinforcement learning framework and implemented partly using a deep neural network. Although the model is proposed as a generic agent model that can simulate a variety of cognitive and decision-making tasks, the model's architecture was originally inspired by an earlier model of the basal ganglia^10^ as shown in Fig. 7A. The representational system is analogous to the cortico-striatal projections that are thought to be capable of compressing the cortical state and generate abstract representations of the same^26^. The memory system is analogous to the striatum proper, and the flip-flops are comparable to the medium spiny neurons (MSNs) of the striatum. The MSNs are known to exhibit UP/DOWN states, a property that is thought to subserve working memory functions^27,28^. In digital systems, flip-flops are used as memory elements that serve as building blocks to implement sequential logic. Thus, in the proposed agent model, the presence of flip-flop neurons in the memory system affords the model the ability to process sequences and perform decision-making functions thereon. The value computation block is analogous to substantia nigra pars compacta (SNc)—it integrates the outputs of the flip-flop neurons of the memory system and computes the value function. The connections from the memory system to the action selection block is analogous to the direct pathway of the basal ganglia. The longer route from the memory system to the explorer block and onward to the action selection block is analogous to the indirect pathway. In modeling literature that describes the decision-making functions of the basal ganglia using reinforcement learning, there is a subclass of models that attribute the role of exploratory drive to the indirect pathway, which is essential to sample the action space randomly^10^. Finally, the action selection block itself is analogous to globus pallidus interna (GPi), the output port of the basal ganglia.

The proposed agent model can also be compared to another brain region known for its decision-making functions—the prefrontal cortex (PFC). Figure 7B shows the analogy between the components of the proposed GRLDNN agent model and areas of PFC whose contributions to decision making have been described extensively^29–32^.

The dorsolateral prefrontal cortex (DLPFC) receives inputs from the primary and secondary sensory association cortices of the posterior brain (Klaus and Pennington 2019). The DLPFC is also considered to be the terminus of the dorsal visual pathway, also called the “where” or “how” pathway that determines how to use visual information by supplying such information to the decision-making mechanisms of the PFC^33^. The DLPFC is also known for its working memory functions, subserved by dopamine-receptor expressing neurons and gated by dopaminergic projections from mesencephalic regions^30^. Thus, the memory system in the proposed agent model is suitably comparable to DLPFC.

Single unit electrophysiological studies have shown the involvement of the orbitofrontal cortex (OFC) in value computation^31^. The role of OFC in value computation was also confirmed by functional imaging studies^29^. Since dopaminergic activity is strongly linked to reward signalling, projections from the ventral tegmental area (VTA) to PFC were implicated in the value computations of OFC^32^. Electroencephalographic studies^34^, on subjects engaged in decision-making activities, have implicated the frontopolar cortex (FPC) in exploratory behavior. Thus, the exploratory block in the proposed model is comparable to FPC. The inferior frontal gyrus^35,36^ is suggested to encode information about NoGo processes and has strong implications for action selection mechanisms, especially action stopping. On the other hand, Anterior Cingulate Cortex (ACC)^37,38^ is suggested to encode information about the uncertainty in choices, hence important for estimating utility values of action choices. Currently only one level of working memory processing is tested in the model. Going forward this aspect will be incorporated where the impact of memory load (by increasing the number of stars and the perceptual levels) can be analysed both experimentally as well as in the model.

The current model also can be made more robust and has the scope of conducting patient profiling. By tuning the appropriate model parameters, we are able to match the experimental results, thereby demonstrating its potential use for profiling real patients. There is a scope to explore further and incorporate aspects of various disease conditions and cognitive disabilities into the model. With respect to the modeling of working memory, we have considered tests at only one difficulty level in our model. There is a scope to scale the model to adopt multiple levels of cognitive loads. The future work also includes integrating the cortical and the subcortical modules into a single framework. In the future, these modeling efforts could also be expanded to include emotion processing and modeling of electrophysiological signals. Altogether, this study opens doors to modeling various cognitive dimensions of the same individual through a unified agent-based modeling framework.

## Supplementary Information

Supplementary Information.

## Data Availability

The raw data supporting the conclusions of this article will be made available by the authors, without undue reservation. Further, inquires can be directed to the corresponding author. The MATLAB code of the proposed GRDLNN model (http://modeldb.yale.edu/267532) is available on the ModelDB server^39^ and an access code will be provided on request.
