# Supplementary material for: A generalized reinforcement learning based deep neural network agent model for diverse cognitive constructs
Source: Sci Rep. 2023 Apr 12;13:5928. doi: 10.1038/s41598-023-32234-y (PMC10097685; doi:10.1038/s41598-023-32234-y)
Supplement: Supplementary file 1 — Supplementary Information. [file 41598_2023_32234_MOESM1_ESM.pdf]

Supplementary Information

A Generalized Reinforcement Learning Based Deep Neural Network Agent Model for Diverse Cognitive Constructs

Sandeep S. Nair[1]; Vignayanandam R. Muddapu[1$]; Vigneswaran C[1]; Pragathi P. Balasubramani[2#]; Dhakshin S. Ramanathan[2,3]; Jyoti Mishra[2]; V. Srinivasa Chakravarthy[1]*

1 Computational Neuroscience Lab, Department of Biotechnology, Bhupat and Jyoti Mehta School of Biosciences, Indian Institute of Technology Madras, Chennai, TN, India.

2 Neural Engineering and Translation Labs, Department of Psychiatry, University of California, San Diego, La Jolla, CA, USA.

3 Department of Mental Health, VA San Diego Medical Center, San Diego, CA, USA.

$ Current address: Blue Brain Project, École Polytechnique Fédérale de Lausanne (EPFL), Campus Biotech, 1202 Geneva, Switzerland.

# Current address: Department of Cognitive Science, Indian Institute of Technology, Kanpur.

* Corresponding Author:

Prof. V. Srinivasa Chakravarthy, Ph.D.

Room 505, Block 1, Department of Biotechnology,

Bhupat and Mehta Jyoti School of Biosciences,

Indian Institute of Technology Madras, Sardar Patel Road,

Adyar, Chennai 600036, Tamil Nadu, India.

Tel: +91 44 2257 4115

Fax: +91 44 2257 4102Email: schakra@ee.iitm.ac.in

S1. The Experimental Tasks

We followed the *BrainE* experimental paradigm results for our simulation study and modeled various tasks used in that framework[1]. Three tasks that were modeled are: *Go Green* (to assess selective attention and response inhibition), *Middle Fish* (to assess distractor processing), and *Lost Star* (to assess working memory) (Figure S 1). Cognitive task details are elaborated on in the sections below.

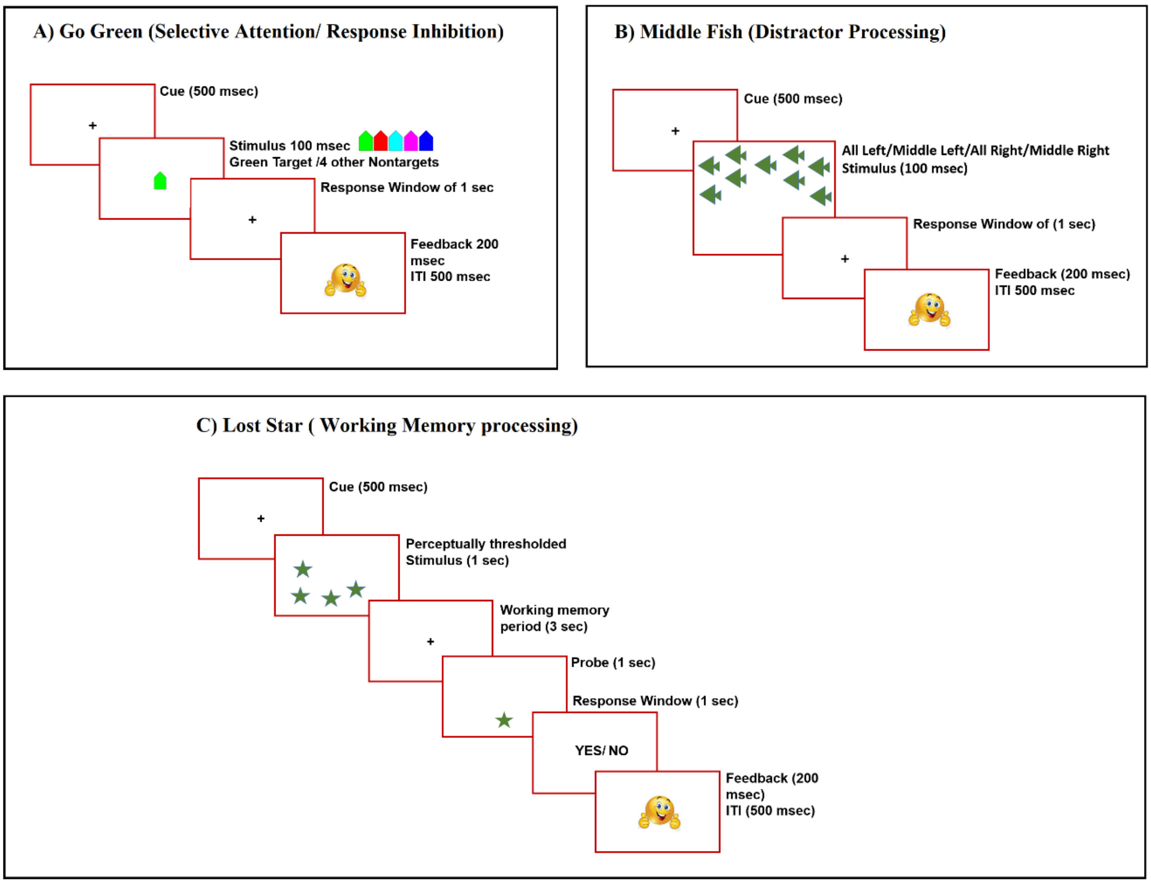

Figure S 1. Overview of the various experimental paradigms used to evaluate selective attention, response inhibition, interference processing, and working memory properties.

1.1. Experimental Paradigm and Tasks

We modeled three *BrainE* platform tasks. The *Go Green* task was used to study both selective attention and response inhibition. The *Middle Fish* task was used to study processing, and the *Lost Star* was used to assess working memory.

Go Green – Selective Attention

Selective attention is tested when the majority of the stimuli are distractors and very few are targets. In this case, one needs to focus on relevant target stimuli and respond as rapidly as possible. For evaluating selective attention, the *Go Green* (Selective attention) task was modeled with the test set consisting of 33% green-colored rocket images and 67% other colored rocket images. Five different colored rocket images were trained with appropriate reward processing based on action selection. In this task, when the image of a green-colored rocket is displayed on the screen, the action 'GO' is selected, whereas for all other colored rocket images, the action 'NO GO' is selected. During each test trial, a cue is presented for an interval of 500 ms (milliseconds), followed by the image of the colored rockets for 100 ms. There is a response window of 1 sec and a feedback window of 200 ms. There is an inter-trial interval (ITI) of 500 ms between each trial and the next.

Go Green – Response Inhibition

Response inhibition is tested when the majority of the stimuli are targets and very few are distractors. In this case, one needs to respond to the stimuli most of the time and focus on the distractors and inhibit their response. For evaluating response inhibition, the *Go Green* task was modeled with a test set consisting of 67% green-colored rocket images and 33% rocket images of other colors. Five different colored rocket images were trained with appropriate reward processing based on action selection. In this task, when the image of a green-colored rocket is displayed on the screen, the action 'GO' is selected, whereas for all other colored rocket images, the action 'NO GO' is selected. During each test trial, at first, a cue is presented for an interval of 500 ms, followed by the image of the colored rockets for 100 ms. There is a response window of 1 sec and a feedback window of 200 ms. Between each trial and the next, there is an ITI of 500ms.

Middle Fish – Distractor Processing

For evaluating distractor processing, the *Middle Fish* task was used. In this task, four different images of fish were used to train the model with appropriate rewards. The image with left-facing (right-facing) fish in the center was rewarded with 1 when the action 'LEFT' ('RIGHT') was selected. Each image consisted of 11 fish, one in the Middle and ten others surrounding the middle one. The decision is made based on the middle fish's orientation (left/right), irrespective of the orientation of the surrounding fish, which act as distractors. During each test

trial, a cue is presented for 500 ms at the start, followed by the array of fish for 100 msec. The images are categorized as all left, middle left, all right, and middle right. All left, and all right images have congruent distractors, while middle left and middle right images have incongruent distractors. To win the reward, for all left and middle left fish images, the action 'LEFT' should be taken, and for all right and middle right, the action 'RIGHT' should be taken. Post-stimulus presentation, a response window of 1 sec, a feedback window of 200 ms, and an ITI of 500 ms are programmed between successive trials.

Lost Star – Working Memory

For evaluating working memory, the *Lost Star* task was used. In this task, a perceptual image needs to be stored in memory for a brief period, and on presentation of the probe image, action needs to be taken based on whether the location of the star in the probe image matches that of one of the stars in the perceptual image. One of the 'YES' or 'NO' actions is selected based on the match. A cue is presented for 500 ms at the start of the test, followed by the perceptual image consisting of four stars at random positions on the screen. This test image is presented for one second, followed by a waiting period of three seconds. Then a probe image consisting of a single star is presented for one second. After this, an action must be selected during the response window of 1 sec, followed by a feedback window of 200 ms. Between successive trials, there is an ITI of 500 ms. Eight levels of this task were carried out[1] depending on the number of stars in the test image. Out of these, in our computational study, we have considered only one level (level 0.5 – four stars in the perceptual image) as there was no comprehensive analysis carried out with respect to variation in levels.

We also consider a few additional tasks from a couple of different works of literature. They are mentioned below.

N-Back – Working Memory

N-back is a continuous working memory task where the subject has to respond with a "yes" when the target stimuli are encountered and respond with a "No" when non-target stimuli are encountered. A representation of the N-back task used in our model is shown in Figure S 2 (A). Target stimuli represent that stimulus, which has also been presented N-timesteps back[2]. The correct response is rewarded. During each test trial, at first, a cue is presented for an interval of

500 ms, followed by the image of the stimuli for 100 ms. There is a response window of 1 sec and a feedback window of 200 ms. Between each trial and the next, there is an ITI of 500ms.

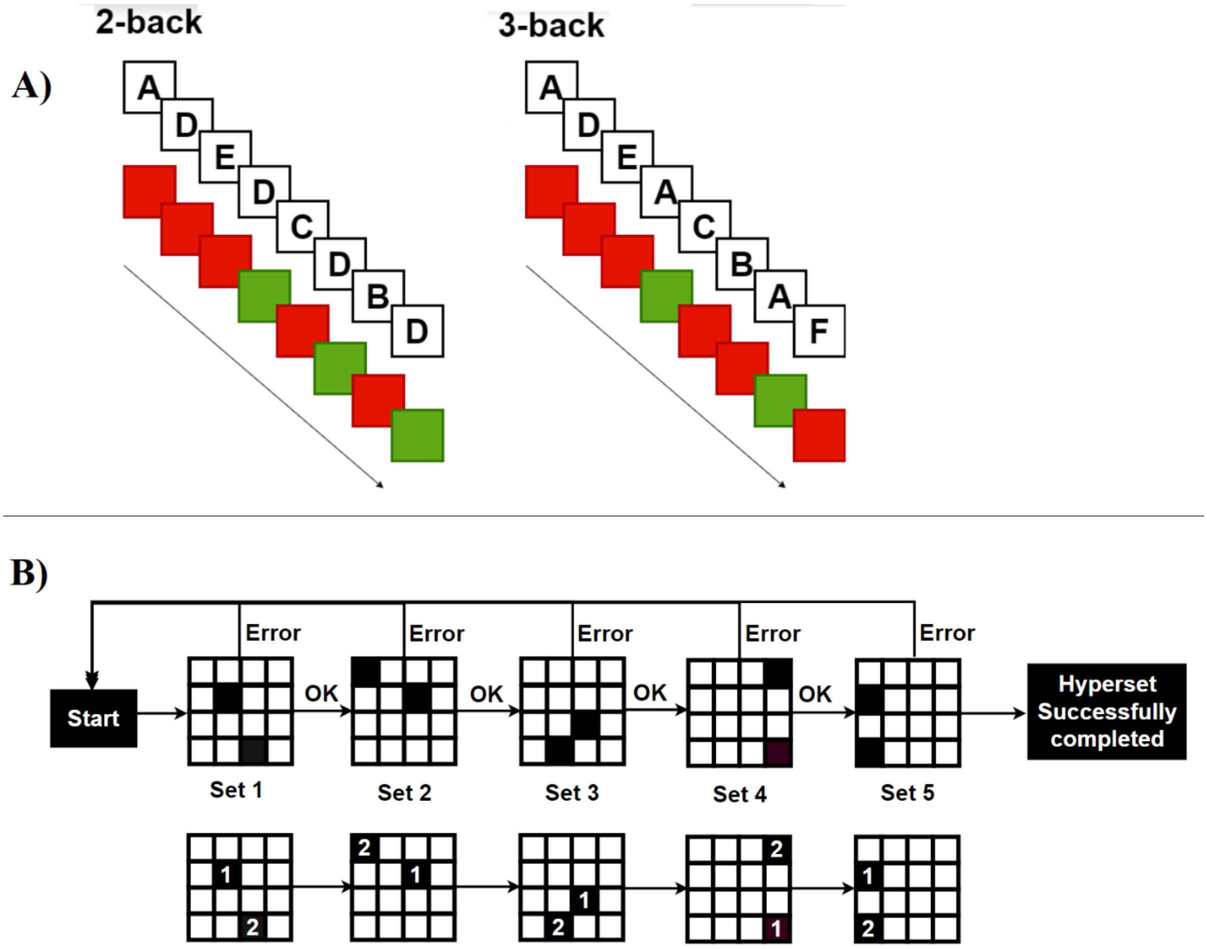

Figure S 2. Overview of the A) N-Back tasks [2] and the B) 2x5 tasks [4] used to evaluate working memory and sequence processing properties.

Sequential 2x5 task

2x5 is a sequential movement task that requires learning and retention of sequences [4]. The input contains a series of five 4x4 grids with two cells highlighted. After presenting each 4x4 grid, the subject has to find the underlying desired two sequential button presses. Each 4x4 input grid is termed a 'set,' and the sequence of five sets is called a 'hyper set.' When any incorrect response is given by the subject, the process is terminated without proceeding to the next set.

Figure S 2(B) depicts the schematic representation of the task. A successful trial requires responding correctly to the hyper set.

T-Maze and Grid-World

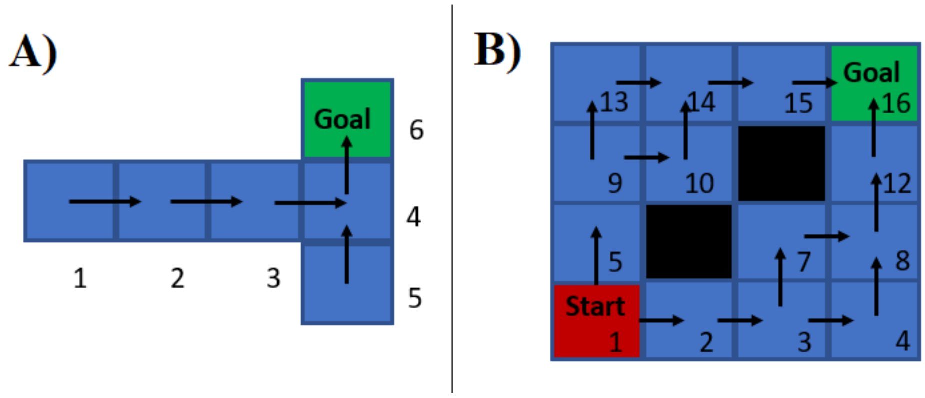

Figure S 3. Overview of the A) T-Maze task and the B) Grid-World task used for the study. The arrows indicate the state transitions. And the black square indicates the obstacles encountered.

We also implemented the tasks (T-maze and Grid world) with state transitions in a discrete action space. For both T-maze and Grid world, the agent starts from a particular start location and traverses through the intermediate states until they reach a goal state as shown in Figure S 3. In the case of a T-maze task, there is comparatively complex decision-making involved near the terminal states, as a wrong choice of action will take the agent further away from the target. In the grid world task, we have introduced the walls in between, and the agent has to avoid bumping into the wall. In both tasks, the reward is given only when the agent reaches the terminal state.

S2. The Architecture

A detailed overview of the model architecture is given in the primary manuscript. As already discussed in the main manuscript, the model has five distinctly identifiable components. 1) Representational System (RS), 2) Memory system (MS), 3) Value Computation System (VC), 4) Explorer Block, and 5) Action Selection System (AS).

S2.1 The Representational System (RS)

The input image is presented in the input layer of the RS module (Figure S 4). Convolutional operation is performed on the input image using 16 feature maps, each using 3x3 kernels. The convolutional layers are followed by max-pooling layers that reduce the feature maps by 2x2. After four such stages of convolution and max-pooling, the resultant outputs were mapped to a fully connected layer of size 64x1 which constitutes the output of the RS encoder module. At the decoder ends, the 1x64 feature output was expanded, followed by deconvolutional layers and pooling layers at the end of which the original image was reconstructed back.

Once the image is perfectly reconstructed, the feature vector output from the fully connected layer of the encoder part of RS is provided as the input to the memory system (MS).

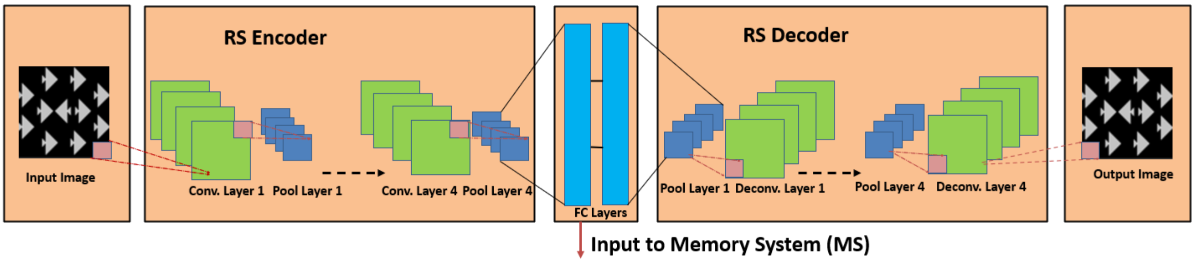

Figure S 4. Representational System (RS), with an input and output layer, four convolutional layers and pooling layers and a fully connected layer at the encoder side. The decoder part consists of a fully connected layers, four pooling and deconvolutional layers. When the input image is satisfactorily reconstructed at the output layer, the encoder output taken from the fully connected layer is passed as input to the Memory System (MS).

S2.1.1 Flip Flop

The neurons of the memory system (MS) is modeled using J-K Flipflops. There are two inputs to the Flipflop (J_{MS1}, K_{MS1}) and there is one output, V^{MS1}. The inputs for the flip-flop neurons of MS1 and MS2 are given by Equations (1-4). The output of the flip flop neuron can be expressed by Equations (5-6) , which is in line with the circuit diagram and the truth table shown in Figure S 5

$$J_{MS1}(t) = W_i^{RS\rightarrow JMS1}(t).X_{RS}(t) \quad (1)$$

$$K_{MS1}(t) = W_i^{RS\rightarrow KMS1}.X_{RS}(t) \quad (2)$$

$$J_{MS2(t)} = W_i^{RS \to JMS2}(t).X_{RS}(t) \quad (3)$$

$$K_{MS2}(t) = W_i^{RS \to KMS2}.X_{RS}(t) \quad (4)$$

$$V_i^{MS1}(t) = J_{MS1}.\left(1 - V_i^{MS1}(t-1)(t-1).\lambda_{MS1}\right) + (1 - K_{MS1}).V_i^{MS1}(t-1).\lambda_{MS1} \quad (5)$$

$$V_i^{MS2}(t) = J_{MS2}.\left(1 - V_i^{MS2}(t-1)(t-1).\lambda_{MS2}\right) + (1 - K_{D2}).V_i^{D2}(t-1).\lambda_{D2} \quad (6)$$

A) J-K Flip Flop Circuit Diagram

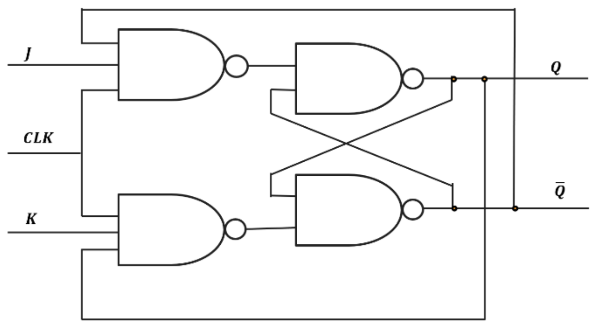

B) Truth Table (JK Flipflop)

J_n	K_n	Q_n	$\overline{Q_n}$	Q_{n+1}	*Action*
0	0	0	1	0	No Change
0	0	1	0	1	No Change
0	1	0	1	0	Reset
0	1	1	0	0	Reset
1	0	0	1	1	Set
1	0	1	0	1	Set
1	1	0	1	1	Toggle
1	1	1	0	0	Toggle

Figure S 5. A) Circuit diagram of JK Flipflop. J, K inputs to the Flipflop; CLK, Clock input; Q, Output of the Flipflop, B) Truth Table of the JK Flipflop If J=K=0, the previous output will be retained, If J=K=1 then the output will be toggled, If (J,K) = (0,1) then the output will be 0, If (J,K) =(1,0) the output will be 1.

S3. Backward Propagation

The weights between the various modules are updated using the backpropagation algorithm. The learning mechanism in this model is broadly divided into three parts. i) MS1-block to AS weight update, and ii) MS1 block to Value Computation (VC) block weight update, iii) RS to MS1/MS2 block weight update. (Figure S 6).

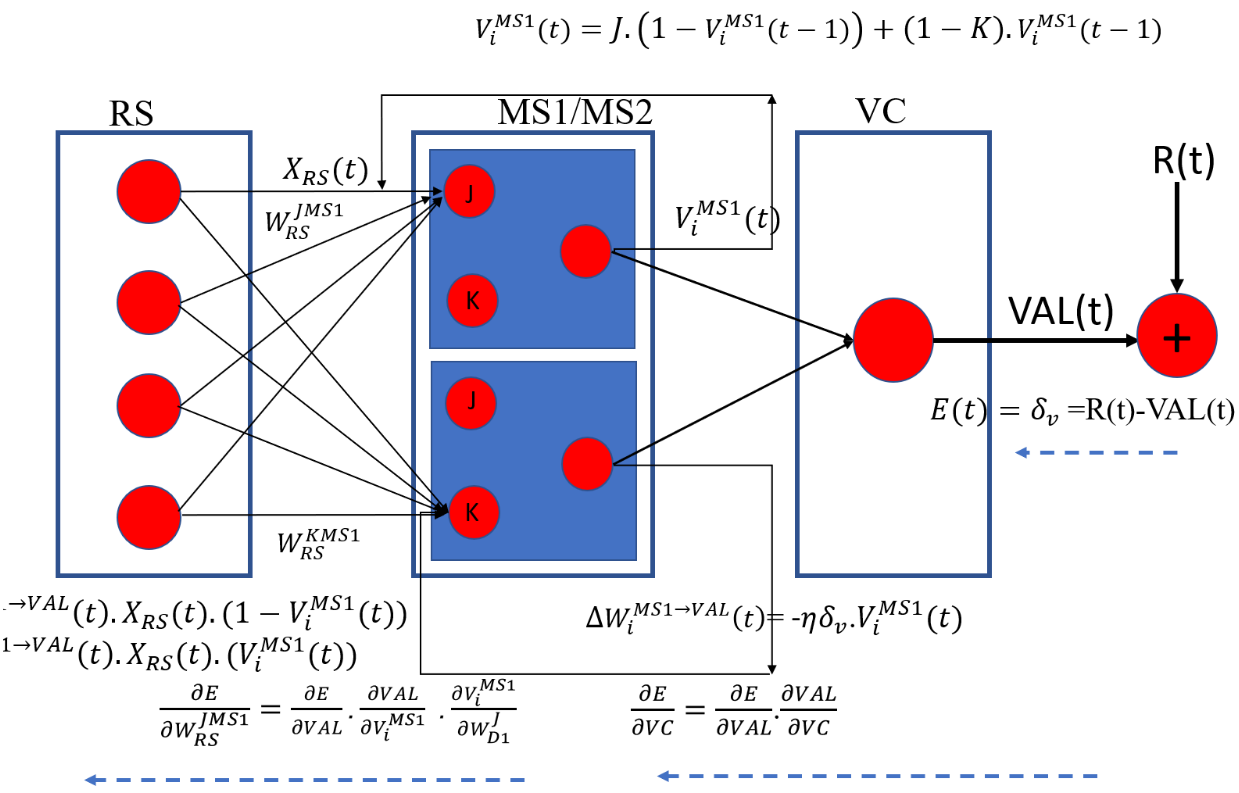

Figure S 6. Schematic diagram representing the forward and backpropagation in the GRLDNN model for the circuit Representative System (RS) –> MS1/MS2 block -> VC. VC is the Value computation block; X_{RS}, is the input from the representation system (RS) block; MS1/MS2 are the sub-blocks of Memory System (MS)block which forwards the data based on the Modulatory signal from VC.

MS1→AS weight update (Q-learning)

The weight update between the MS1 and the AS blocks is governed by Q-learning. The Q table is updated based on the expected reward and the actual reward. The expected reward is high (1) when for a set of given states, the desired action is selected. For example, in case of Go-Green task the Go action is desired when a green rocket is presented, and No-Go action is desired when other coloured rockets are presented. Action Selection block AS is modeled as a race model. Based on the inputs and the D1-AS weights an action is selected out of the possible two. Depending on the action the actual reward will be either 1 or 0. Based on the Expected and the actual rewards the weights are further updated using back propagation using Q-Learning.

The temporal difference error δ_q is used to update the weights between the neurons of the D1 block and the AS block, as shown in Equations (7 to 9). $\delta_q$is calculated using the Equation 7. Here r(t) is the reward obtained for selecting the particular action at that time instant, $Q_t(s,a)$ is the Q value obtained for a particular state action pair and $Q_{t+1}(s,a)$ is the future rewards. A discount factor for the future reward given by γ is applied to the value that maximizes all possible future actions. The gradient in weight between D1 and AS is found by multiplying this temporal difference error with the output of the D1 block and applying a learning rate η_q as shown in Equation 8.

$$\delta_q(t) = r(t) + \gamma * \max_a Q_{t+1}(s,a) - Q_t(s,a) \tag{7}$$

$$\Delta W_i^{D1\to AS} = -\eta_q * \delta_q * V_i^{D1} \tag{8}$$

$$\Delta W_i^{D1\to AS} = W_i^{MS1\to AS} + \Delta W_i^{MS1\to AS} \tag{9}$$

MS1 - block → VC weight update:

Cortico-striatal weights are updated using $\Delta W_i^{D1\to VAL}$, which is the gradient of the error, E, with respect to the weights between D1 and M blocks. The Error, E, is defined as the difference between the reward obtained for the particular action selected and the Value function as mentioned in Equation. 11. The computation of value function is already shown in Equation. 11 of main manuscript, where the output of the D1 block combines over the connections with respective weights given by $W^{D1\to M}$ in order to obtain the Value function (VF).

$$r(t) = \sum_{i=1}^{n} r_{i^t} * AS_i^t \tag{10}$$

$$E = \delta_V = r(t) - VAL(t) \tag{11}$$

$$\Delta W_i^{MS1\to VAL} = -\eta_V * \delta_V * V_i^{MS1} \tag{12}$$

$$W_{(i,t+1)}^{MS1\to VAL} = W_i^{MS1\to VAL} + \Delta W_{(i,t)}^{MS1\to VAL} \quad (13)$$

$$r(t) = \sum_{i=1}^{n} r_{i^t} * AS_i^t \quad (14)$$

RS → MS1/MS2 blocks weight update

The MS1/MS2 sub-blocks that comprise the MS are modeled using the Flip Flop neurons which facilitates the delay and memory properties.

In order to compute the gradients for the weights between the input block and the JK ports of D1/D2 block, let us refer to Figure S 6 The Error, E, in the output that is the mean squared error between the reward received and the value function is propagated backwards through the module M. Here $W_i^{I\to JD1}, W_i^{I\to KD1}, W_i^{I\to JD2} W_i^{I\to KD2}$ are the weights between the input block, I, and the input J/K ports of the Flip Flops at MS1/MS2 module.

$$\Delta W_i^{RS\to JMS1} = \frac{\partial E}{\partial W_i^{RS\to JMS1}} = \frac{\partial E}{\partial VAL} \cdot \frac{\partial VAL}{\partial V_i^{MS1}} \cdot \frac{\partial V_i^{MS1}}{\partial W_i^{RS\to JMS1}} \quad (15)$$

$$\Delta W_i^{RS\to KMS1} = \frac{\partial E}{\partial W_i^{RS\to KMS1}} = \frac{\partial E}{\partial VAL} \cdot \frac{\partial VAL}{\partial V_i^{MS1}} \cdot \frac{\partial V_i^{MS1}}{\partial W_i^{RS\to KMS1}} \quad (16)$$

$$\Delta W_i^{RS\to JMS2} = \frac{\partial E}{\partial W_i^{RS\to JMS2}} = \frac{\partial E}{\partial VAL} \cdot \frac{\partial VAL}{\partial V_i^{MS2}} \cdot \frac{\partial V_i^{MS2}}{\partial W_i^{RS\to JMS2}} \quad (17)$$

$$\Delta W_i^{RS\to KMS2} = \frac{\partial E}{\partial W_i^{RS\to KMS2}} = \frac{\partial E}{\partial VAL} \cdot \frac{\partial VAL}{\partial V_i^{MS2}} \cdot \frac{\partial V_i^{MS2}}{\partial W_i^{RS\to KMS2}} \quad (18)$$

$$\frac{\partial VAL}{\partial V_i^{MS1}} = W_i^{MS1\to VC} \quad (19)$$

$$\frac{\partial V_i^{MS1}}{\partial W_i^{RS\to JMS1}} = (1 - V_i^{MS1}) \tag{20}$$

Combining Equations (11,19,20) we can rewrite the Equation (15-18) as Equation (21) and Equation (23-25) respectively.

$$\Delta W_{JMS1} = \frac{\partial E}{\partial W_i^{RS\to JMS1}} = -\delta_V . W_i^{MS1\to VAL} . (1 - V_i^{MS1}) \tag{21}$$

$$\frac{\partial V_i^{MS1}}{\partial W_i^{RS\to KMS1}} = -V_i^{MS1} \tag{22}$$

$$\Delta W_{JMS2} = \frac{\partial E}{\partial W_i^{RS\to JMS2}} = -\delta_V . W_i^{MS2\to VAL} . (1 - V_i^{MS2}) \tag{23}$$

$$\Delta W_{KMS1} = \frac{\partial E}{\partial W_i^{RS\to KMS1}} = -\delta_V . W_i^{MS1\to VAL} . (-V_i^{MS1}) \tag{24}$$

$$\Delta W_{KMS2} = \frac{\partial E}{\partial W_i^{RS\to KMS2}} = -\delta_V . W_i^{MS2\to VAL} . (-V_i^{MS2}) \tag{25}$$

S3.1 Performance Assessment

The model performance is assessed in terms of the metrics of accuracy, reaction time, speed, consistency, and efficiency, as defined below.

- *Mean Reaction time (RT)* is defined as the number of seconds taken for the winning neuron to cross the threshold.

$$RT = \frac{1}{N}\sum_{i=1}^{n} RT_i$$

- *Speed* is measured as the logarithm (to the base 10) of the inverse of the reaction time. It indicates the speed of decision-making.

$$Speed = Log_{10}(\frac{1}{(RT)})$$

- *Consistency* provides the measure of the consistency of the performance speed.

$$\text{Consistency} = 1- \frac{\sqrt{\sum_{i=1}^{n}(RT_i - RT)^2}}{N}$$

- *Dprime Accuracy, d'* = z(Hits)-z(False Alarms) (theoretical max 4.65) is the signal detection sensitivity normalized to a 0-1 scale
- *Efficiency* is a composite metric of speed and accuracy; higher efficiency is achieved when there is less speed accuracy trade-off.

The below section shows the effect of parameter tuning on performance.

S3.2 Selection of Learning Parameters and Model parameter tuning

In our current study, we studied the performance efficiency of our model with respect to the variations in the lateral connectivity strength and the threshold parameter. The lateral connectivity strength in the explorer module and the threshold for the race model were the primary contributing factors involved in learning. The explorer model is configured using a variant of Vanderpol oscillators known as the Lienard system of oscillators, which will induce randomness into the circuit based on the input and the lateral and interconnectivity weights. Two oscillatory subsystems-one excitatory and the other inhibitory, are connected back-to-back. Hence, we studied the efficiency majorly concerning these two parameters. Here we did not consider the interconnectivity weights to reduce complexity because it has a comparatively lesser influence. The threshold parameter is set at the action selection block, configured using rate-coded neurons. The action selection block is configured as a race model, and whichever action neurons cross the threshold is selected as the winner. Apart from this, when we expand our model to introduce disease conditions, we will be using the delta parameter, which is analogous to the dopamine component.

S4. Results

S4.1 Training Phase

During the learning progresses, the value functions keep increasing and approaches the value of 1, which is the maximum value that can be attained. Figure S 7 represents the state value functions for various tasks with Figure S 7A representing the Go Green (Selective Attention) task, Figure S 7B representing the Go Green (Response Inhibition) task, Figure S 7C representing the Middle Fish (Distractor Processing) task and Figure S 7D representing the Lost Star (Working Memory) task. The Q values for the respective states and action pair during corresponding to the correct action is seen to be higher whereas the value corresponding to the wrong action is observed to be lower. Figure S 8 represents the Q value at the end of training for the A) Go Green (Selective Attention), B) Go Green (Response Inhibition), C) Middle Fish (Distractor Processing) and D) Lost Star (Working Memory processing).

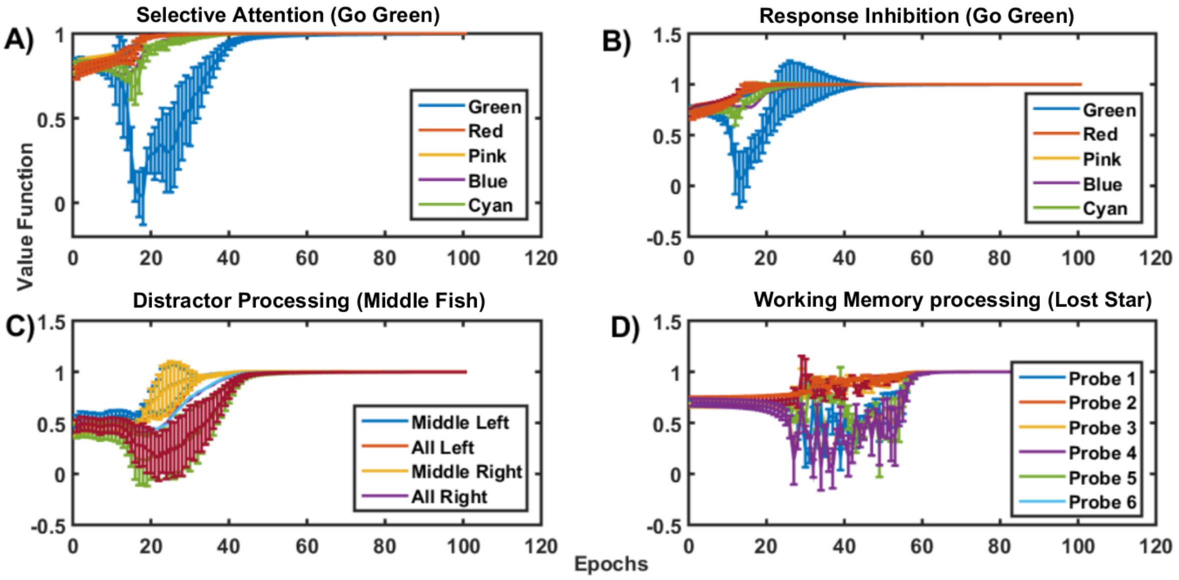

Figure S 7. A) The state value functions over the epochs during the training phase for four different tasks as represented in A) The Go Green (Selective Attention) B) The Go Green (Response Inhibition) C) The Distractor Processing task (Middle Fish) and D) The working Memory Task (Lost Star).

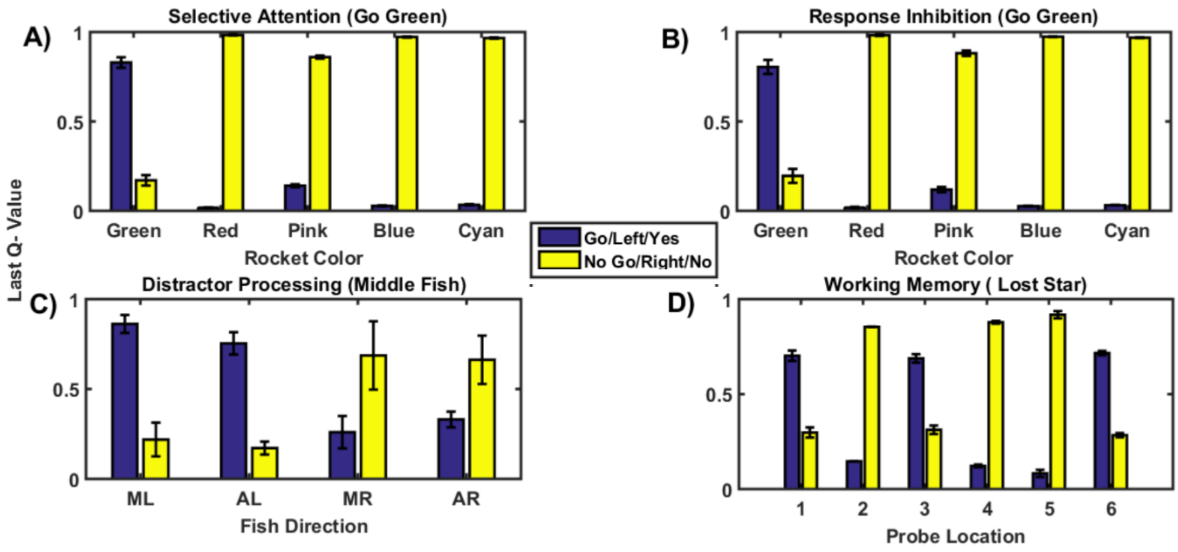

Figure S 8. A) The Q values corresponding to the state action pair at the end of the training phase for four different tasks as represented in A) The Go Green (Selective Attention) B) The Go Green (Response Inhibition) C) The Distractor Processing task (Middle Fish) and D) The working Memory Task (Lost Star) is shown. The Q value corresponding to the correct action should be higher and wrong action should be lower. Accordingly, in A and B the Q value corresponding to Go action when green colored rocket is presented is higher and in C the Q value corresponding to the Left action is higher. In case of D, the Q value corresponding to 'Yes' action is higher when the star location in the probe image matches that of the perceptual image. The blue bar represents the actions Go/Left/Yes and the yellow bar represents the actions No Go/Right/No.

S4.1.1 N-Back and 2x5 Task Results

The model results of the N-back are comparable and relatable to the performance results[2], while the 2x5 task shows the evaluation of a different type of task using the same modeling paradigm, whose results are comparable to the experimental results[3,4]. The performance of the N-Back task were already discussed in the main manuscript. Figure S 9 shows the performance of the 2x5 task.

The representation of the N-Back task simulated is shown in Figure S 2 (A).

2x5 is a sequential movement task that requires learning and retention of sequences[3,4]. The input contains a series of five 4x4 grids with two cells highlighted. After presenting each 4x4 grid, the subject has to find the underlying desired two sequential button presses. Each 4x4 input grid is termed a 'set,' and the sequence of five sets is called a 'hyper set.' When any incorrect response is given by the subject, the process is terminated without proceeding to the next set. Figure S 2 (B) depicts the schematic representation of the task as a successful trial requires responding correctly to the hyper set. Figure S 9 (A) shows the number of trials taken to achieve ten successful trials across the number of days, whereas Figure S 9 (B) shows the

number of successful trials across the number of trials performed for day 1, day 3, day 10, and day 30 respectively.

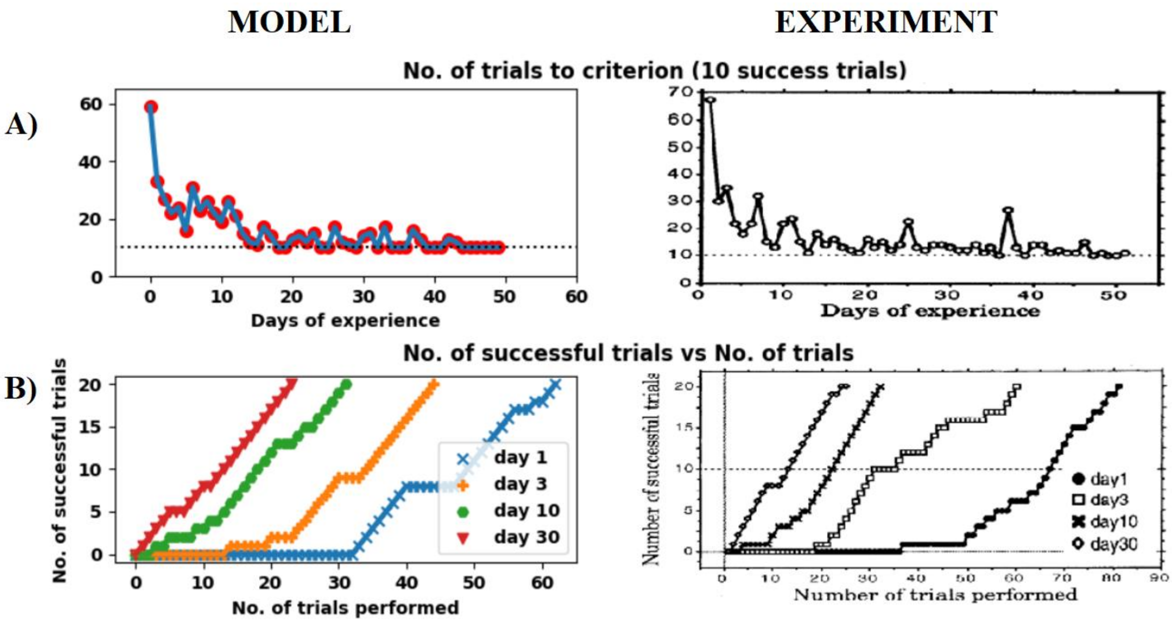

Figure S 9. A) The figure shows the number of trials taken to achieve 10 successive trials. The figure on the left-hand side indicates the Model results and the one on the right hand side indicate the experimental results B) The figure shows the number of successful trials versus the number of trials performed. The figure on the left hand side indicates the Model results and the one on the right hand side indicate the experimental results.

We also implemented the tasks (T-maze and Grid world) with state transitions in a discrete action space. For both T-maze and Grid world, the agent starts from a particular start location and traverses through the intermediate states until they reach a goal state. In the case of a T-maze task, comparatively complex decision-making is involved near the terminal states, as a wrong choice of action will take the agent further away from the target. In grid world task we have introduced the walls in between, and the agent has to avoid bumping into the wall. In both the tasks the reward is given only when the agent reaches the terminal state.

S4.2 Markov Decision Model for Experiments

The Markov decision model for the experiments is given below. From the reinforcement learning point of view, the tasks implemented Go-Green, Middle Fish, and Lost Star, are primarily the Go-No-Go tasks with a definite state-action relationship; however, no state transitions depending on actions. Figure S 10 shows the state action relationship for the Go-

Green task. Depending on the stimulus, either an action, Go or No Go, is selected, and the reward depends upon the accuracy of the agent's action. The state action relationships are maintained in a Q-Table and are as shown in the Table 1 below.

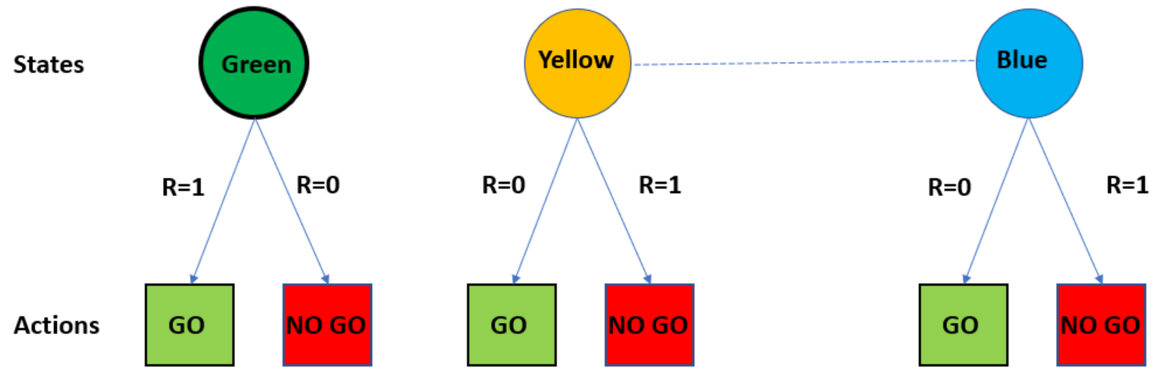

Figure S 10. The state action pairs of the Go-Green task is shown in the figure. For the green stimuli action Go is preferred where as for the other colored tasks action 'No-Go' is preferred. Reward is given for the correct response.

Table 1. The state action pairs of the Go-Green task is shown . The rows represent the actions and the columns represents the states.

Action\State	Green	Yellow	Red	Cyan	Blue
GO	H	L	L	L	L
NO GO	L	H	H	H	H

The working memory task involves composite state space based on the initial stimulus and the subsequent matching stimulus (Figure S 11). However, here too, we do not have any state transitions depending on the action taken.

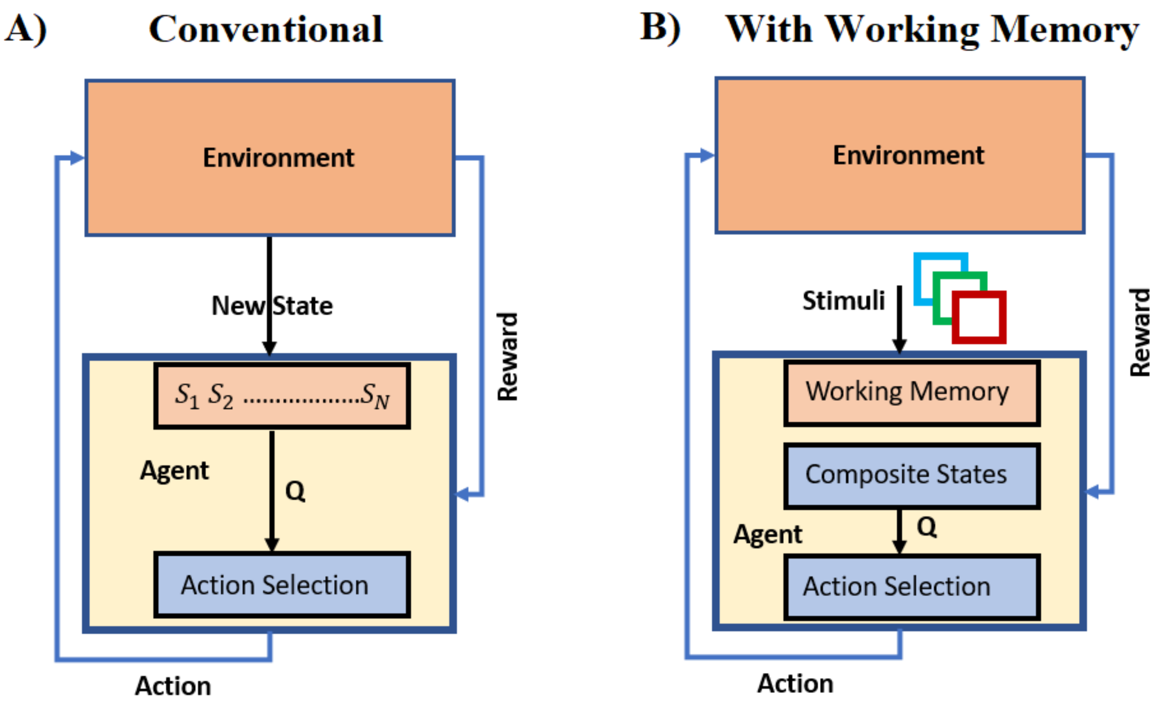

Figure S 11. A) Representation of a conventional RL system with interactions between the Agent and the Environment, **B)** Representation of an RL system with working memory functionality and the interactions between the Agent and the Environment

We also implemented the tasks (T-maze and Grid world) with state transitions in a discrete action space. For both T-maze and Grid world, the agent starts from a particular start location and traverses through the intermediate states until they reach a goal state. In case of a T-maze task there is a comparatively complex decision making involved near the terminal states as a wrong choice of action will take the agent further away from the target. In the grid world task, we have introduced the walls in between, and the agent has to avoid bumping into the wall. In both tasks, the reward is given only when the agent reaches the terminal state. The state transitions for the T-Maze task are shown in Figure S 12.

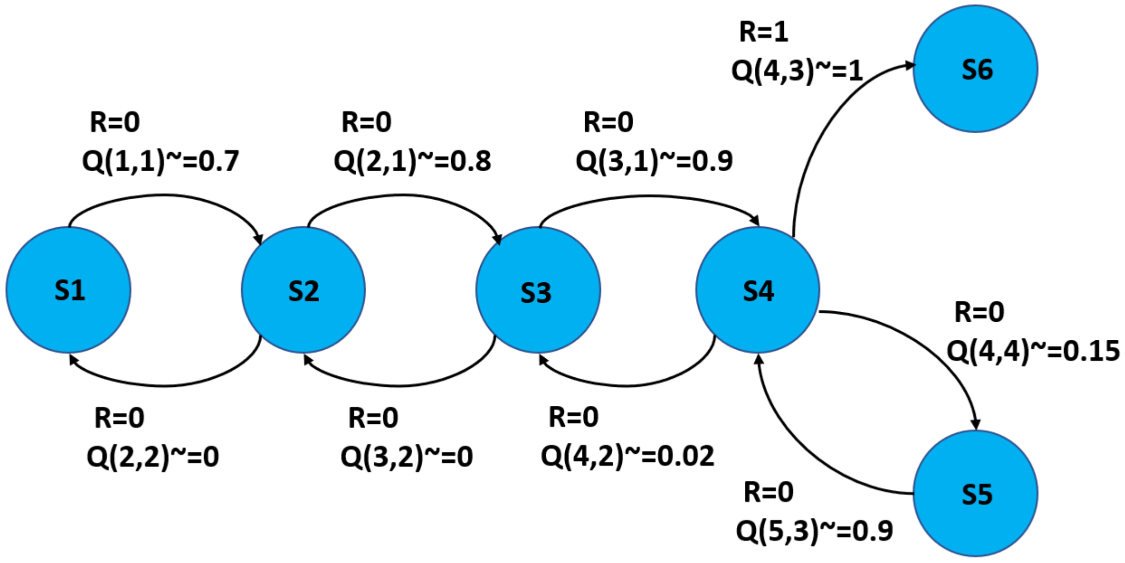

Figure S 12. **A)** Representation of Markov Decision process involved in a T-maze task. The reward(R) is given only at the goal state (S6). With each transition of the agent towards the goal state the Q-table is updated. Actions Right, Left, Up and Down are represented by numeric 1,2,3 and 4 respectively. States 1,2,3,4,5 and 6 corresponds to the positions of the T-Maze task as shown in Figure S 3 (A).

S4.3 Stability and Convergence of Q Networks

In our current study, the three tasks were modeled as per the experimental paradigm considered. The tasks Go-Green, Middle-Fish and Lost-Star where involved small discrete state space and there were no action based state transitions. For these tasks we did not encounter any convergence or stability issues. However, with more complex networks involving large state space and action dependent state transitions, we do observe convergence and stability issues. We have considered two tasks, T-maze and Grid-world, that involved state transitions. To achieve faster convergence, we maintained a separate Q-Target network, which was updated periodically after every few epochs. For Q-networks involving large state space and continuous state spaces the stability issues arises where the Q values never converges. In that case the typical workarounds adopted are maintaining a target Q-network. The target Q-network lags behind the main Q-network and the parameters of the target Q-network is not trained, however are periodically updated from the main-Q network.

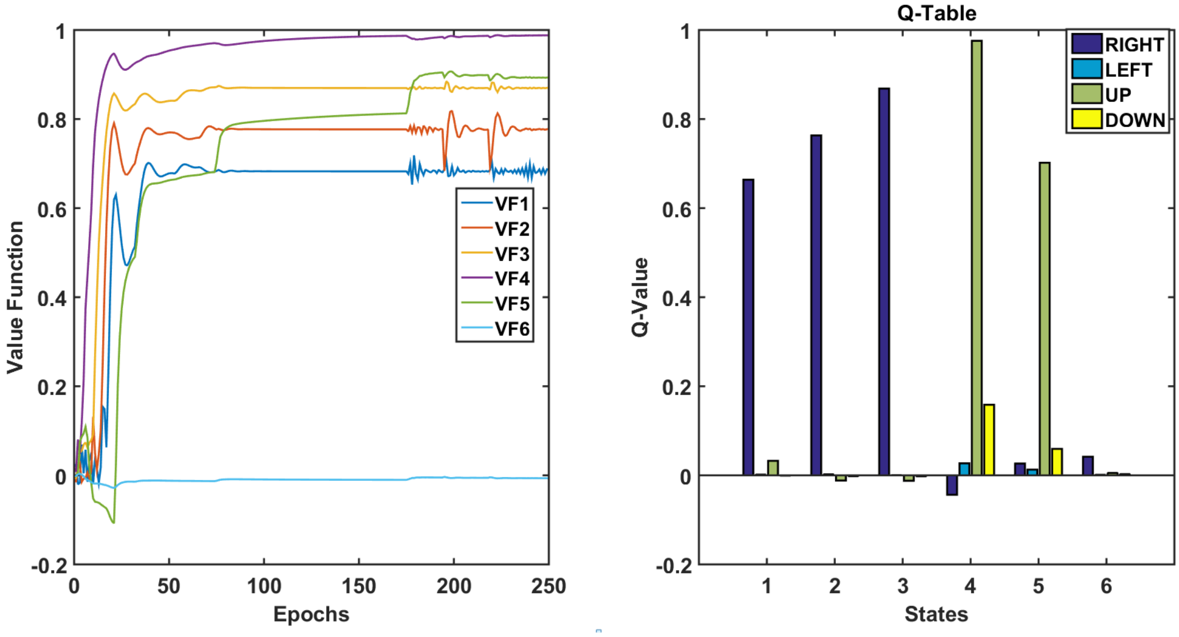

Figure S 13. A) The state value functions over the epochs during the training phase for the six discrete states in the T-maze task B) The last updated Q-table indicating the state-action values. States are indicated in the x-axis and the Q-values are updated in the y-axis

.

Figure S 13 shows the state value function over the epochs during the training phase and the final Q value at the end of training. VF1,VF2,VF3,VF4,VF5 and VF6 represents the value function for states 1 to 6 respectively(Figure S 13 (A)). As can be seen in Figure S 3(A), state 4 is close to the goal state and expectedly we can see that the value function for state 4 approaches to 1. State 3, state 2 and state 1 attains comparatively lesser values in the decreasing order as state 3 is the immediate previous state to state 4 and state 2 is the immediate previous state to state 3 and state 1 is the immediate previous state to state 2. (Figure S 13 (B) shows the last updated Q values. For each state there are four set of actions (Right, Left, UP and Down). From Figure S 3(A), we can see that when the agent performs the action ‘UP’ from state 4 the goal state is reached and a reward is received. Hence at state 4 the action ‘UP’ attains the highest value.

S4.4 Model parameters and Average Performance

We selected the model parameters t $V_{TH} = 0.4 \; and \; \epsilon = 0.05$, as the RMSE values were found to be lowest with these parameters when the model performance was compared with that of the average performance of the experimental results. The RMSE values for the four tasks with model parameters $V_{TH} = 0.4 \; and \; \epsilon = 0.05$, are as given in the Table 2 below.

Table 2. RMSE for different tasks compared to average experimental performance

TASK	(Average Speed)			
	Model (Esilon =0.05, Threshold=0.4)	Desired (Exp)	MSE	RMSE
Go Green (SA)	0.351	0.358	4.9E-05	0.007
Go Green (RI)	0.3756	0.3976	0.000484	0.022
Middle Fish	0.3211	0.3139	5.18E-05	0.0072
Lost Star	0.1468	0.0626	0.00709	0.0842

S4.3 Model parameter Estimation

We modeled the mapping between the experimental parameters (speed, consistency) and the model meta-parameters (ϵ , V_{TH}), tuning using a simple multilayer perceptron model (MLP). Given the input values of speed and consistency, we predict the corresponding values of ϵ and V_{TH} by using a multi-layered perceptron model consisting of one input layer, two hidden layers, and an output layer as shown in Figure S 14. The input layer consisted of 2 neurons whereas each hidden layer consisted of 32 neurons each and after each hidden layer 'RELU (Rectified Linear Unit)' activation was used. The output layer consisted of two neurons followed by a sigmoid activation function. The model output gives the predicted values of the meta parameters (ϵ and V_{TH}). Hence by varying the values of the two meta-parameters, we would be able to calibrate the GRLDNN model in order to simulate the experimental performance. Twelve data points consisting of different combinations of (ϵ and V_{TH}) used to simulate respective speed and consistency values were used for training, and the model was trained for 50000 epochs with the training error in the order of ~10^{-5} at the end of training. The predicted and desired values of both the threshold and epsilon were closely matched.

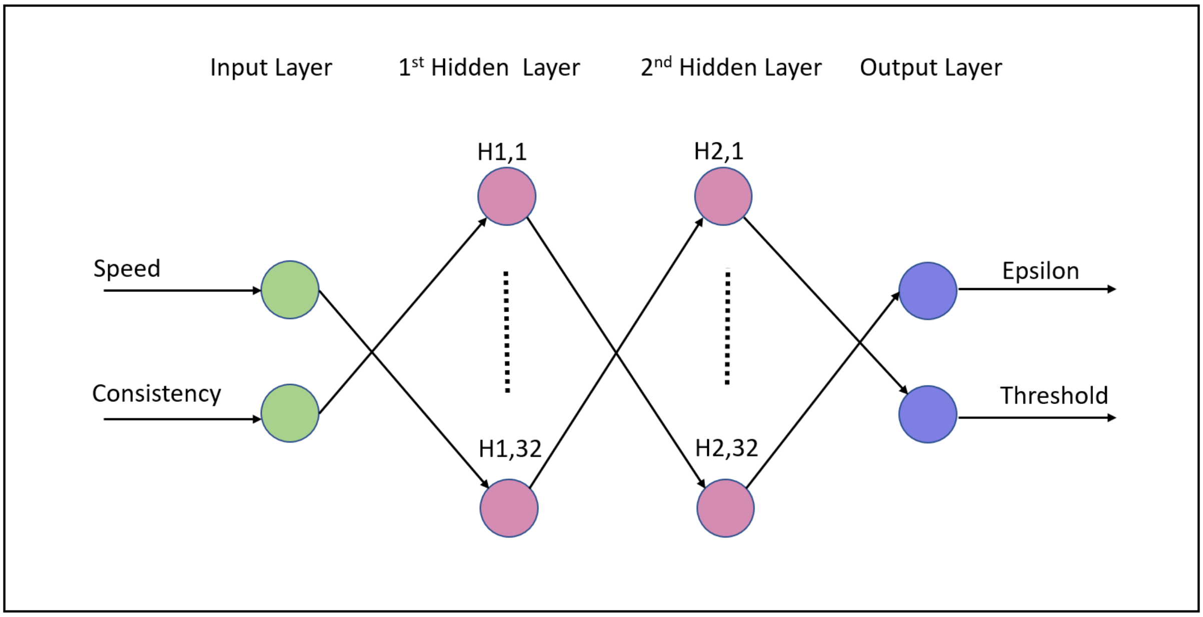

Figure S 14. Multi-layered perceptron model to predict the meta parameters with one input layer, two hidden layers and one output layer. Speed and consistency are given as inputs to the model and Epsilon and threshold are predicted. H1,1 to H1,32 represents the neurons in first hidden layer and H2,1 to H2,32 represents the neurons in the second hidden layer.

S4.4 Correlation Between Tasks

The empirical performance results of the cognitive tasks indicate strong correlations between selective attention, response inhibition, and distractor processing cognitive functions. This is partly by design, as all of these tasks were set up as speeded stimulus-response tasks. The working memory task showed a very weak correlation with the performance of other tasks. This could very well relate to the differences between the goals of the working memory task, to identify the probe as to whether it matches with a test template but not a particularly speeded response to the probe. Working memory processing also differs from the other core functions of selective attention, response inhibition, and distractor processing in terms of distributed recruitment of brain activation[5–7].

S5. RL Models of higher order learnings in humans

Multi-arm Bandits is one of the RL tasks that can be used to evaluate higher order learning in humans[8]. This involves problem solving and decision making. The task requires selecting the optimum action with a trade-off between taking an action based on our existing knowledge of

the environment and exploring in order to update our knowledge base and thereby increasing the chances of a reward in the future. The task is related to variety of cognitive processes including memory, learning, attention and executive functions and are linked to various disorders such as depression, addiction and other neurological disorders. Similarly Iowa Gambling task is another RL task that is used to access the impulsive decision making[9–11]. These models can throw light on the underlying neural mechanisms responsible for decision making behaviour of people with diverse cognitive profile. However, these models are just simplified representations of human cognition, and may not be able to capture all aspects of real-world decision-making.

Various studies have shown that the n-back tasks performance is closely related with intelligence, attention, and working memory[2,6,12–14]. The n-back tasks are be used to test various hypothesis related to working memory. When it comes to higher order cognitive characteristics such as attention and inhibitory control Go-No Go tasks are quite useful. The task provides an evaluation about how accurately and quickly the response to the target is and the suppression of response to non-target stimulus is. The T-maze task is another RL task which involves decision making where the agent has traverse multiple states and has to choose one of the actions left or right at the junction and the task requires ability to store and retrieve information and take actions based on the rules. In the grid world task, the agent has to navigate towards the goal avoiding the obstacles and is a much more complex task compared to the-maze and includes the aspects of attention, working memory, goal oriented behaviour and navigation

The task set we have modeled replicating the BrainE experimental tasks covers the cognitive aspects such as the Attention, Distractor processing, Inhibitory control, Working memory, processing speed and executive functions.

S6. Fragmented and Integrative approaches

Much of the research on cognition has followed a fragmented approach, where individual cognitive processes are studied in isolation. There is a long history of this kind of research in psychology and neuroscience, with different fields of study focusing on specific aspects of cognition such as attention, memory, perception, emotions, language, and reasoning[15–19]. However, by focusing on the individual cognitive functions, it is possible to miss interrelated features, complex brain functions, and their diversity. It will also be challenging to interpret and integrate findings from multiple studies. Different characteristics of cognitive disorders

and the commonalities among these various characteristics of these disorders have been summarized[20].

Our attempt is to integrate multiple aspects of cognition into a unified framework. We elaborate that many simple cognitive paradigms are mostly studied in silos experimentally[21–25] and computationally[26–32]. A recent study by our group tested the efficiency of a battery of simple experimental cognitive tasks (on attention, distractibility, working memory, and emotion processing) and showed it is complex enough to robustly explain human behavior across age and mental health spectrum[1,33]. We note that one aim of our modeling effort is to simulate behavior across age and mental health spectrum, and we start with modeling the tasks described in the BrainE battery. To summarize, the fragmented approach results in potential limitations such as lack of integration, simplification of complexity, overgeneralization, narrow focus, and difficulty in interpretation.

S7. Performance Metrics for cognitive assessment

Our modeling study has simulated the results of the experimental data obtained from[1], which focuses on the human ability to focus on relevant stimuli of immediate perceptual goals, suppress irrelevant information, and store and retrieve relevant information. In general, the performance of these cognitive tasks is accessed by the ability to achieve the goal with less reaction time with minimal errors. Several studies have used this metric to analyze cognitive performance[2]. The reaction time variation and accuracy variation are reliably estimated by introducing a composite metric as discussed in previous works[15,34]. We have included 'efficiency' as a performance metric in this line. The consistency indicates the reliability of the performance over different repetitions of the experiment. It is measured by variance in speed, which is inversely correlated with reaction time. We will update this paragraph in the supplementary section.

S8. References

1. Balasubramani, P. P. *et al.* Mapping cognitive brain functions at scale. *Neuroimage* **231**, (2021).

2. Lamichhane, B., Westbrook, A., Cole, M. W. & Braver, T. S. Exploring brain-behavior relationships in the N-back task. *Neuroimage* **212**, (2020).

3. Nakahara, H., Doya, K. & Hikosaka, O. Parallel cortico-basal ganglia mechanisms for acquisition and execution of visuomotor sequences - A computational approach. *J Cogn Neurosci* **13**, (2001).

4. Hikosaka, O., Rand, M. K., Miyachi, S. & Miyashita, K. Learning of sequential movements in the monkey: Process of learning and retention of memory. *J Neurophysiol* **74**, (1995).

5. Smith, E. E. & Jonides, J. Storage and executive processes in the frontal lobes. *Science* vol. 283 Preprint at https://doi.org/10.1126/science.283.5408.1657 (1999).

6. RepovŠ, G. & Baddeley, A. The multi-component model of working memory: Explorations in experimental cognitive psychology. *Neuroscience* **139**, (2006).

7. Konstantinou, N., Beal, E., King, J. R. & Lavie, N. Working memory load and distraction: Dissociable effects of visual maintenance and cognitive control. *Atten Percept Psychophys* **76**, (2014).

8. Daw, N. D., Gershman, S. J., Seymour, B., Dayan, P. & Dolan, R. J. Model-based influences on humans' choices and striatal prediction errors. *Neuron* **69**, (2011).

9. Bechara, A., Damasio, A. R., Damasio, H. & Anderson, S. W. Insensitivity to future consequences following damage to human prefrontal cortex. *Cognition* **50**, (1994).

10. Mandali, A., Chakravarthy, V. S., Rajan, R., Sarma, S. & Kishore, A. Electrode Position and Current Amplitude Modulate Impulsivity after Subthalamic Stimulation in Parkinsons Disease—A Computational Study. *Front Physiol* **7**, 585 (2016).

11. Aram, S. *et al.* The Iowa Gambling Task: A Review of the Historical Evolution, Scientific Basis, and Use in Functional Neuroimaging. *Sage Open* **9**, (2019).

12. Kane, M. J., Conway, A. R. A., Miura, T. K. & Colflesh, G. J. H. Working Memory, Attention Control, and the N-Back Task: A Question of Construct Validity. *J Exp Psychol Learn Mem Cogn* **33**, (2007).

13. Jaeggi, S. M. *et al.* The relationship between n-back performance and matrix reasoning - implications for training and transfer. *Intelligence* **38**, (2010).

14. Redick, T. S. & Lindsey, D. R. B. Complex span and n-back measures of working memory: A meta-analysis. *Psychon Bull Rev* **20**, (2013).

15. Barlow, H. B. The absolute efficiency of perceptual decisions. *Philos Trans R Soc Lond B Biol Sci* **290**, (1980).

16. Deco, G. & Rolls, E. T. Attention, short-term memory, and action selection: A unifying theory. *Progress in Neurobiology* vol. 76 Preprint at https://doi.org/10.1016/j.pneurobio.2005.08.004 (2005).

17. Buschman, T. J. & Miller, E. K. Top-down versus bottom-up control of attention in the prefrontal and posterior parietal cortices. *Science (1979)* **315**, (2007).

18. Chun, M. M. & Turk-Browne, N. B. Interactions between attention and memory. *Current Opinion in Neurobiology* vol. 17 Preprint at https://doi.org/10.1016/j.conb.2007.03.005 (2007).

19. Hickok, G. & Poeppel, D. The cortical organization of speech processing. *Nature Reviews Neuroscience* vol. 8 Preprint at https://doi.org/10.1038/nrn2113 (2007).

20. Millan, M. J. *et al.* Cognitive dysfunction in psychiatric disorders: Characteristics, causes and the quest for improved therapy. *Nature Reviews Drug Discovery* vol. 11 Preprint at https://doi.org/10.1038/nrd3628 (2012).

21. Sternberg, S. High-speed scanning in human memory. *Science (1979)* **153**, (1966).

22. Eriksen, B. A. & Eriksen, C. W. Effects of noise letters upon the identification of a target letter in a nonsearch task. *Percept Psychophys* **16**, (1974).

23. Greenberg, L. M. & Waldmant, I. D. Developmental Normative Data on The Test of Variables of Attention (T.O.V.A.™). *Journal of Child Psychology and Psychiatry* **34**, (1993).

24. Bechara, A. *Iowa Gambling Task Professional Manual*. *Psychological Assessment Resources, Inc* (2007).

25. Lin, C.-H., Chiu, Y.-C. & Huang, J.-T. Gain-loss frequency and final outcome in the Soochow Gambling Task: A Reassessment. *Behavioral and Brain Functions* **5**, (2009).

26. Lavie, N., Hirst, A., de Fockert, J. W. & Viding, E. Load theory of selective attention and cognitive control. *J Exp Psychol Gen* **133**, (2004).

27. Navalpakkam, V. & Itti, L. Modeling the influence of task on attention. *Vision Res* **45**, (2005).

28. Frank, M. J., Loughry, B. & O'Reilly, R. C. Interactions between frontal cortex and basal ganglia in working memory: A computational model. *Cognitive, Affective and Behavioral Neuroscience* vol. 1 Preprint at https://doi.org/10.3758/CABN.1.2.137 (2001).

29. Braver, T. S., Gray, J. R. & Burgess, G. C. Explaining the Many Varieties of Working Memory Variation: Dual Mechanisms of Cognitive Control. in *Variation in Working Memory* (2012). doi:10.1093/acprof:oso/9780195168648.003.0004.

30. Hampshire, A. & Sharp, D. J. Contrasting network and modular perspectives on inhibitory control. *Trends in Cognitive Sciences* vol. 19 Preprint at https://doi.org/10.1016/j.tics.2015.06.006 (2015).

31. Chakravarthy, V. S. & Moustafa, A. A. *Computational Neuroscience Models of the Basal Ganglia*. *Movement disorders : official journal of the Movement Disorder Society* vol. 15 (Springer Singapore, 2018).

32. Houk JC, Adams JL, B. A. A Model of How the Basal Ganglia Generate and Use Neural Signals That Predict Reinforcement. in *Models of Information Processing in the Basal Ganglia* (2018). doi:10.7551/mitpress/4708.003.0020.

33. Grennan, G. *et al.* Cognitive and Neural Correlates of Loneliness and Wisdom during Emotional Bias. *Cerebral Cortex* **31**, (2021).

34. Vandierendonck, A. A comparison of methods to combine speed and accuracy measures of performance: A rejoinder on the binning procedure. *Behav Res Methods* **49**, (2017).
